# Microbial predictors of healing and short-term effect of debridement on the microbiome of chronic wounds

**DOI:** 10.1038/s41522-020-0130-5

**Published:** 2020-05-01

**Authors:** Samuel Verbanic, Yuning Shen, Juhee Lee, John M. Deacon, Irene A. Chen

**Affiliations:** 1 Program in Biomolecular Science and Engineering, University of California, Santa Barbara, CA USA; 2 Department of Chemistry and Biochemistry, University of California, Santa Barbara, CA USA; 3 Department of Statistics, University of California, Santa Cruz, CA USA; 4 Goleta Valley Cottage Hospital, Ridley-Tree Center for Wound Management, Santa Barbara, CA USA; 5 Department of Chemical and Biomolecular Engineering, University of California, Los Angeles, CA USA

**Keywords:** Clinical microbiology, Microbiome

## Abstract

Chronic wounds represent a large and growing disease burden. Infection and biofilm formation are two of the leading impediments of wound healing, suggesting an important role for the microbiome of these wounds. Debridement is a common and effective treatment for chronic wounds. We analyzed the bacterial content of the wound surface from 20 outpatients with chronic wounds before and immediately after debridement, as well as healthy skin. Given the large variation observed among different wounds, we introduce a Bayesian statistical method that models patient-to-patient variability and identify several genera that were significantly enriched in wounds vs. healthy skin. We found no difference between the microbiome of the original wound surface and that exposed by a single episode of sharp debridement, suggesting that this debridement did not directly alter the wound microbiome. However, we found that aerobes and especially facultative anaerobes were significantly associated with wounds that did not heal within 6 months. The facultative anaerobic genus *Enterobacter* was significantly associated with lack of healing. The results suggest that an abundance of facultative anaerobes is a negative prognostic factor in the chronic wound microbiome, possibly due to the increased robustness of such communities to different metabolic environments.

## Introduction

Chronic wounds are wounds that fail to exhibit reasonable healing progress within an expected time frame (e.g., 3–6 weeks)^1,2^. It is estimated that in the U.S. alone, over 6.5 million people are affected, costing the healthcare system at least $25 billion annually^3^. Although the burden of chronic wounds is often overlooked or obscured by overall burden of the primary disease^1,2^, these wounds have a notable impact on quality of life, reducing mobility, and inducing chronic pain. Older patients with established diseases, particularly diabetes, obesity, venous insufficiency, peripheral artery disease, and immobility, are at highest risk of developing chronic wounds^3^. As these risk factors increase in prevalence, the economic and human costs of chronic wounds are expected to grow.

One of the leading impediments to healing of chronic wounds is infection and associated pathological inflammation^4^. Although chronic wounds are not always infected, they may be colonized by a distinct microbiome that could lead to infection or impact wound healing. While traditional, culture-dependent studies are now acknowledged to be unable to provide an extended view of diversity, more recent culture-independent studies over the past decade have established that wounds harbour diverse microbiota, with the primary constituents being *Staphylococcus* spp*.*, *Pseudomonas* spp*.*, *Corynebacterium* spp*.*, *Streptococcus* spp*.*, *Anaerococcus* spp., and *Enterococcus* spp., along with numerous low-abundance taxa^5–7^. While chronic wounds are polymicrobial, they have lower diversity than healthy skin^8^. Substantial inter-patient variability exists in the microbiome, which cannot be explained by age, race, sex, or wound etiology^5,9^, and therefore statistical models that can account for inter-patient variability are desirable for modeling the chronic wound microbiome. Despite significant past work^5–27^, additional studies on the wound microbiome are needed to understand its contribution, if any, to the pathophysiology of chronic wounds. Here, we investigate how a single episode of sharp debridement affects the wound microbiome, as well as which constituents of the wound microbiome might correlate with healing.

A second leading impediment to wound healing is biofilm formation^4,28^. One of the most common and widely effective chronic wound treatments is debridement, a standard-of-care procedure whose goal is physical disruption and removal of biofilms and necrotic or devitalized tissue^29,30^. Besides stimulating reepithelialization and cell migration, debridement can reduce microbial load^29,30^. However, relatively little is known about how debridement influences the composition of the microbial community of the wound. Previous work has found that microbiota isolated from debrided tissue and wound swabs are similar though not an exact match^8^. A recent longitudinal diabetic foot ulcer study found that, after 2 weeks, debridement had significantly decreased the relative abundance of anaerobes, but only in the wounds that healed within 12 weeks^7^. To determine whether this response occurred immediately vs. developed over the 2-week interval, we swabbed chronic wounds immediately after sharp debridement in the same clinic visit and compared the microbial communities before and after debridement, including a comparison of the abundance of individual taxa.

An important focus of study for the chronic wound microbiome is the identification of correlations of the microbiome to healing outcomes. For example, Loesche et al.^6^ determined that temporal instability of communities, particularly the transition between several distinct community types, is associated with positive healing outcomes. Understanding which organisms are beneficial or detrimental could be important for evaluating prognosis or probiotic interventions. However, no specific taxa or metabolic types have yet been reported to be predictive of healing outcomes. We studied whether the presence of taxa with different oxygen requirements (aerobes, anaerobes, and facultative anaerobes) or specific taxa predicted healing outcomes 6 months after wound sampling.

In the present study, wound swabs were obtained from 20 patients presenting at a wound clinic, with 5 patients from each of four common chronic wound etiologies (diabetic, venous, arterial, and pressure ulcers). Swab samples were collected from chronic wounds before and after a single, sharp debridement event, along with a skin swab sample from a control site (e.g., the contralateral limb). Microbial communities were characterized by Illumina sequencing of the V1–V3 loops of 16S rRNA genes. Data were analyzed by ecological diversity metrics and differential abundance analysis with DESeq2^31^, a popular differential abundance method, and a Bayesian generalized linear mixed regression model (BGLMM) with patient-specific factors to account for inter-patient variabilities^32^. Our analysis of debridement indicates that the newly exposed wound surface has minimal microbial difference from the old wound surface, and we identify bacterial taxa associated with healing outcomes. The implications of these findings on our understanding of the pathophysiology of chronic wounds is discussed.

## Results

### Bacterial composition of skin and chronic wound microbiomes

Patient and wound characteristics are summarized in Table 1. A total of 18,128,419 paired-end sequencing reads were obtained from Illumina sequencing, with 14,025,888 reads assigned in demultiplexing. On average, there were 203,273 reads in each sample (minimum = 15,476 reads, maximum = 729,495 reads, median = 172,250 reads). Quality control analysis indicated sufficient sampling of the microbiome in all but one sample, which was excluded from analysis (Supplementary Fig. 1).

**Table 1:** Patient and wound characteristics.

Patient #	Wound type	Healing outcome	Wound size (sq. cm)	Level of debridement	Instrument	# Previous debridements	Days since last debridement
1	Diabetic	Healed	1	Dermis	Curette	0	0
2	Diabetic	Unhealed	0.5	Dermis	Curette	12	14
3	Diabetic	Healed	3.57	Dermis	Curette	5	8
4	Diabetic	Unhealed	68.7	Subcutaneous	Curette	6	7
5	Diabetic	Unhealed	3.6	Subcutaneous	Curette	7	10
6	Venous	Unhealed	2.07	Dermis	Curette	33	7
7	Venous	Unhealed	30	Dermis	Curette	1	7
8	Venous	Healed	11.6	Dermis	Curette	4	9
9	Venous	Healed	445	Dermis	Curette	2	7
10	Venous	Healed	10	Subcutaneous	Curette	3	9
11	Arterial	Healed	0.2	Dermis	Curette	6	9
12	Arterial	Unhealed	307.84	Dermis	Curette	18	7
13	Arterial	Unhealed	5.92	Dermis	Curette	13	7
14	Arterial	Healed	6.4	Subcutaneous	Curette	13	7
15	Arterial	Unhealed	10.85	Dermis	Curette	2	7
16	Pressure	Healed	0.2	Subcutaneous	Tissue Nipper	3	12
17	Pressure	Unhealed	8.88	Dermis	Curette	20	7
18	Pressure	Healed	0.9	Dermis	Curette	19	6
19	Pressure	Unhealed	4.62	Subcutaneous	Scalpel	4	7
20	Pressure	Unhealed	1.35	Dermis	Curette	3	7

**Fig. 1:**
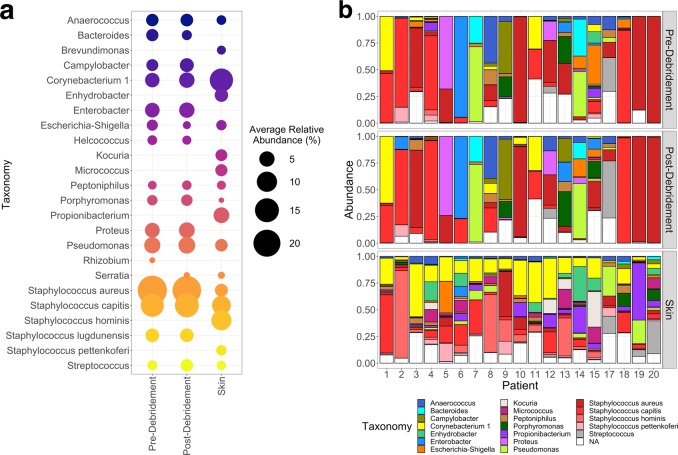
Taxonomic composition of skin and wound samples. **a** Average relative abundance of genera within each sample type (only genera with average relative abundance >1% are shown). *Staphylococcus* taxa are labeled at the species level. Gradient color scale is for visualization purposes only. **b** Relative abundance of genera in each sample (bar graph limited to the 20 most abundant taxa overall; “NA” indicates OTUs without taxonomic classification; *Staphylococcus* taxa are labeled at the species level).

We first verified that our results on the skin and wound microbiomes of the patients were consistent with previous findings^7,20^. Sequenced 16S rRNA genes were clustered into operational taxonomic units (OTUs) using the open OTU picking method in QIIME^33^, with taxonomy assigned using the SILVA128 database^34^ (see Supplementary Fig. 1 for quality metrics). The accuracy of microbial community recapitulation was confirmed by analysis of a cell-based mock community. All expected members of the mock community were detected, but some deviations from the expected composition were observed (Supplementary Fig. 2). In particular, a relative decrease of Gram-positive organisms compared to Gram-negative organisms suggests that incomplete lysis may cause relative under-representation of Gram-positive organisms in the samples. Negative control samples were analyzed to identify potential contaminants (Supplementary Table 1). Compositional data were obtained (i.e., relative abundance within each sample) and absolute abundance was not measured specifically. However, we noted that the absolute concentration of DNA extracted from negative control samples was undetectable by a Qubit assay but that nearly all skin and wound samples (59/60) resulted in detectable DNA^35^, indicating that absolute abundances are generally higher in skin and wound samples compared to the negative controls. The four most abundant phyla detected on average across both skin and wound samples were *Firmicutes*, *Proteobacteria*, *Actinobacteria*, and *Bacteroides* (Supplementary Fig. 2). On skin, the most abundant genera were, in decreasing order, *Staphylococcus*, *Corynebacteria*, *Propionibacteria*, *Pseudomonas*, *Micrococcus*, *Enhydrobacter*, and *Kocuria* (Fig. 1). Although these data were not ideal for giving species-level resolution, due to the important role of *Staphylococcus* species in skin infections, *Staphylococcus* OTUs were further tentatively assigned to species based on alignment of the V1–V3 loops. Skin samples contained diverse communities of *Staphylococcus* species, with *S. hominis* and *S. capitis* the most abundant members on average. In wound samples (both pre- and post-debridement), *Staphylococcus* was also the most abundant genus, and *Corynebacteria* and *Pseudomonas* were also major constituents. Other major constituents of the wound samples included *Proteus*, *Enterobacter*, *Campylobacter*, *Porphyromonas*, *Streptococcus*, *Bacteroides*, and *Anaerococcus* (Fig. 1). Similar to skin samples, wound samples contained diverse *Staphylococcus* species, including *S. capitis*, though *Staphylococcus aureus* was the most abundant on average.

These results confirm earlier findings^5–7,36^ and verify the reliability of our samples. See Supplementary Figs. 3 and 4 and Supplementary Note 1 for more details.

### Differences in abundance of individual bacterial taxa in skin vs. chronic wound microbiomes

**Fig. 2:**
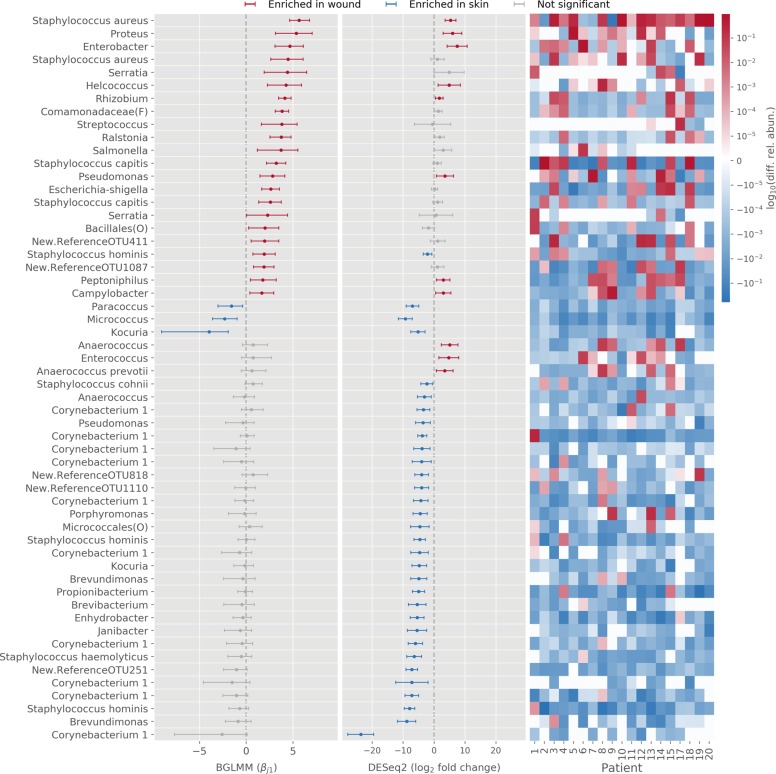
Association of abundant OTUs with pre-debridement wound samples or skin samples, inferred by DESeq2 or BGLMM. OTUs (with average relative abundance > 0.1%) found to be significant (criteria described in Methods) in at least one of the models with enrichment in wound samples (red) or enrichment in skin samples (blue). OTUs found to be not significantly enriched in that model are shown as gray. For DESeq2, the log_2_ fold-change in variance-stabilized abundance is shown with error bars indicating the estimated 95% confidence interval (1.96× standard error, *n* = 19). For BGLMM, the median of estimated $\beta _{j1}$ (pre-debridement effect for OTU $j$, see Methods for details) with 95% credible interval error bars are reported (*n* = 19). The heatmap shows the log_10_ (relative abundance in wound minus relative abundance in skin) of each OTU of each patient for a visual comparison. OTUs are labeled by their genus name or lowest available taxonomy assignment if applicable; otherwise, the original OTU label from QIIME open OTU picking is used. Note that multiple OTUs may belong to the same genus.

The individual taxa overrepresented in skin or wounds are of interest for identifying potential keystone species or biomarkers of the healthy vs. diseased state. Several taxa appear to differ in abundance between skin and wounds (Fig. 1, Supplementary Fig. 5). To determine the significance of such observations, we used DESeq2 and BGLMM to identify statistically significant associations of individual OTUs with wounds (pre-debridement) or skin. DESeq2 estimates the confidence interval of the log fold-change in abundance of each OTU between skin and wound samples, assuming count data follow a negative binomial distribution with dispersion estimated by combining data across OTUs^31^. Analyzing a filtered OTU table (OTUs present in >5 samples with >10 counts; Supplementary Fig. 1) using DESeq2, 97 out of 462 OTUs had significant differential abundance between skin and wounds (adjusted *p* value < 0.05). Of these, 25 OTUs were enriched in wounds and 72 were enriched on skin. We focus on “abundant” OTUs having average relative abundance >0.1% (11/25 of OTUs enriched in wounds and 32/72 of OTUs enriched in skin) (Fig. 2). To corroborate the DESeq2 analysis, we applied the Bayesian model BGLMM and used posterior credible intervals to identify significant associations. We validated BGLMM using simulations (Supplementary Note 2). Applied to our data, BGLMM recapitulated the observed OTU counts reasonably well (Spearman’s correlation coefficient > 0.75; Supplementary Fig. 6). BGLMM found 54 OTUs with significant associations (i.e., 95% credible intervals not including zero), with 50 being enriched in pre-debridement samples (22 being abundant) and only 4 being enriched in skin samples (3 being abundant) (Fig. 2).

Despite some discrepancies between the two models, several abundant OTUs were identified by both analyses. For example, both models identified *S. aureus*, *Proteus*, *Enterobacter*, *Helcococcus*, and *Pseudomonas* genera as strongly enriched in wound (pre-debridement) samples, and *Paracoccus*, *Micrococcus*, and *Kocuria* as significantly enriched in skin samples. Compared to the qualitative description (Fig. 1), we validated that some OTUs from highly abundant genera (>1% relative abundance) that appeared to be exclusive to either wound or skin samples indeed were statistically significantly associated with either wound or skin. In particular, *Proteus*, *Enterobacter*, *and Helcococcus* were both exclusive to and significantly enriched in wound samples, while *Kocuria* and *Micrococcus* were both exclusive to and significantly enriched in skin samples. These associations and the variability among patients can be visually validated in the accompanying heat map (Fig. 2). In both models, significant OTUs comprise roughly half of the total abundance across the samples (Supplementary Fig. 7).

### Minimal changes to the chronic wound microbiome immediately after debridement

The pre- and post-debridement wound microbiome samples were found to have similar diversity (Supplementary Fig. 3), and visual inspection of the community composition (Fig. 1b) suggests a high degree of similarity before and after debridement. However, the average unweighted UniFrac distance between pre- and post-debridement samples was substantial (0.42 ± 0.09) (Supplementary Fig. 4), although the average weighted UniFrac distance (0.086 ± 0.059) was much smaller (Supplementary Fig. 4). This pattern indicates that the major taxa are largely unchanged by debridement, but that there may be changes to the low-abundance taxa. This feature can be observed in the ordination analysis (Supplementary Fig. 4), in which the pre- and post-debridement samples from each patient appear to cluster with each other in the weighted UniFrac and Bray-Curtis ordinations but not in the unweighted UniFrac ordination.

**Fig. 3:**
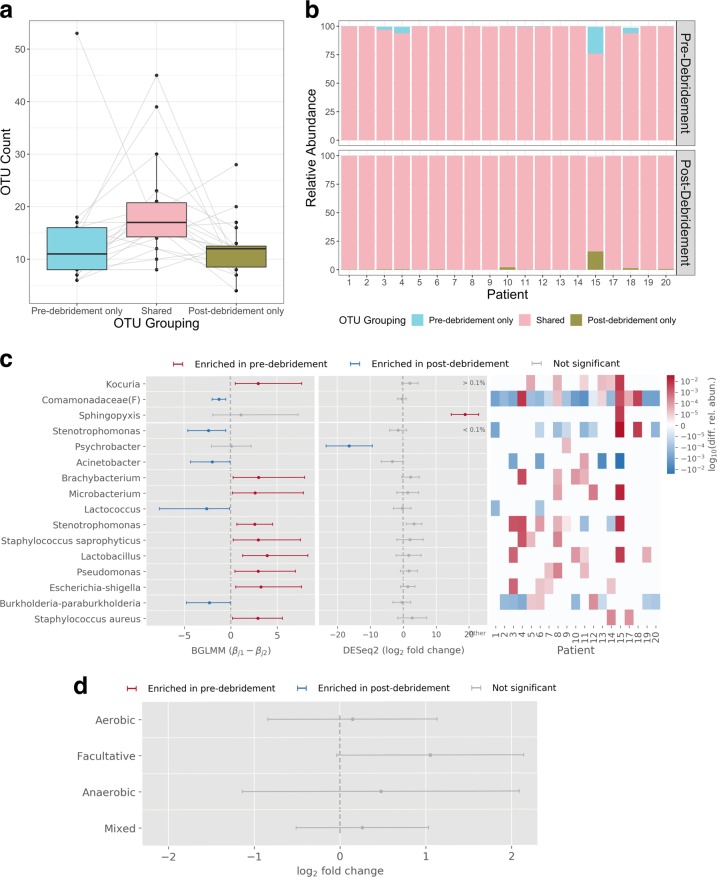
Comparison of pre- and post-debridement samples. Pre- and post-debridement samples have similar numbers of exclusive OTUs (**a**); lower and upper bounds of the boxes correspond to the first and third quartiles, center lines indicate the median, and whiskers extend up to 1.5× interquartile range; any points beyond the whiskers are outliers. Shared OTUs account for a large majority of microbiota (**b**). **c** Analysis of statistically significant enrichment of individual taxa in pre- vs. post-debridement samples by DESeq2 and BGLMM; OTUs are sorted by descending average relative abundance. Note that *Sphingopyxis* was only found to be abundant in patient 15. **d** Coarse-grained differential abundance analysis of aerobes, anaerobes, and facultative anaerobes using DESeq2 shows no significant difference immediately after debridement. “Mixed” indicates taxa that were not annotated due to: low relative abundance (<0.1% on average), no taxonomic annotation, or ambiguous oxygen requirements. For all BGLMM and DESeq2 inferences, error bars indicate 95% confidence interval, or 1.96× standard error, respectively, and *n* = 19.

To better understand the difference between pre- and post-debridement samples, we identified the OTUs in each patient that were unique to either the pre- or post-debridement communities vs. present in both. On average, a similar number of OTUs were found to be unique to pre-debridement samples (13.8 ± 11.4) or unique to post-debridement samples (12.0 ± 5.3), while 19.4 ± 9.3 OTUs were shared between the two (Fig. 3a). Consistent with the UniFrac metrics, OTUs unique to either pre- or post-debridement samples constituted a small proportion of overall composition (2.04 ± 5.52% and 1.17 ± 3.66% on average, respectively) while shared OTUs accounted for the vast majority (98.4 ± 4.64%) of the sample composition on average (Fig. 3b).

To determine whether individual OTUs were affected by debridement, regardless of uniqueness, we used DESeq2 and BGLMM to identify which OTUs were significantly associated with pre- or post-debridement samples (Fig. 3c). For OTUs with >0.1% relative abundance, *Kocuria* (strict aerobes and facultative anaerobes) and *Sphingopyxis* (strict aerobes) may be enriched in pre-debridement samples while the *Comamonadaceae* family may be enriched in post-debridement samples. However, each association was detected by only one method, limiting overall confidence in these inferences.

Since debridement was previously noted to affect anaerobes in particular after 2 weeks^7^, we further characterized the oxygen requirements of the OTUs unique to pre- or post-debridement samples that also had an average relative abundance greater than 0.1%. Note that post-debridement samples in this study were taken in the same clinic visit as pre-debridement samples, i.e., immediately after debridement. OTUs unique to pre-debridement samples, included ten aerobes (0.99% average relative abundance), six anaerobes (0.44% average relative abundance), and five facultative anaerobes (0.37% average relative abundance). OTUs unique to post-debridement samples contained seven aerobes (0.39% average relative abundance), two anaerobes (0.28% average relative abundance), and two facultative anaerobes (0.55% average relative abundance). Although the number of taxa unique to pre- or post-debridement samples was small, the findings suggested a slight decrease of anaerobes post-debridement. To further probe whether anaerobes were immediately depleted by debridement, we grouped OTUs into the following four categories according to oxygen requirements: aerobes, anaerobes, facultative anaerobes, and taxa containing a mixture of these. None of these types showed a statistically significant difference between pre- and post-debridement samples using DESeq2 (Fig. 3d). Together these findings suggest that debridement by itself does not lead to an immediate alteration in the oxygen-requirement types comprising the wound microbiome, and changes, if any, to the taxonomic composition are likely to be small.

### Healing and nonhealing wounds exhibit similar immediate response to debridement

Chronic wounds were categorized into two groups based on whether the wounds had healed by 6 months after sampling (when medical record abstraction occurred for consented patients). Eight wounds fell in the “healed” category and 12 in the “non-healing” category. The age of the unhealed wounds was therefore known to be >6 months. Wound age was estimated using the time of first presentation as a proxy for the start of the wound. For healed wounds, wound age was known for 4 out of 8 patients; of those, 2 healed in <12 weeks of treatment and 2 healed after 6–9 months. A previous study found that chronic wounds that healed within 12 weeks, but not wounds that did not heal within 12 weeks, showed a significant drop in Shannon diversity 2 weeks after debridement^7^. In our samples, no significant change in bacterial diversity of the pre- and immediately post-debridement wound swabs was observed for either healing outcome (Supplementary Fig. 8), suggesting that the previously observed drop in diversity reflects a gradual shift in the microbiome of healing wounds. Similarly, the microbiomes of healing and non-healing wounds did not differ in UniFrac distances to skin, indicating that the microbiomes of healing wounds did not exhibit a statistically significant overall similarity to the skin microbiome at this time point, compared to non-healing wounds (Supplementary Fig. 7).

**Fig. 4:**
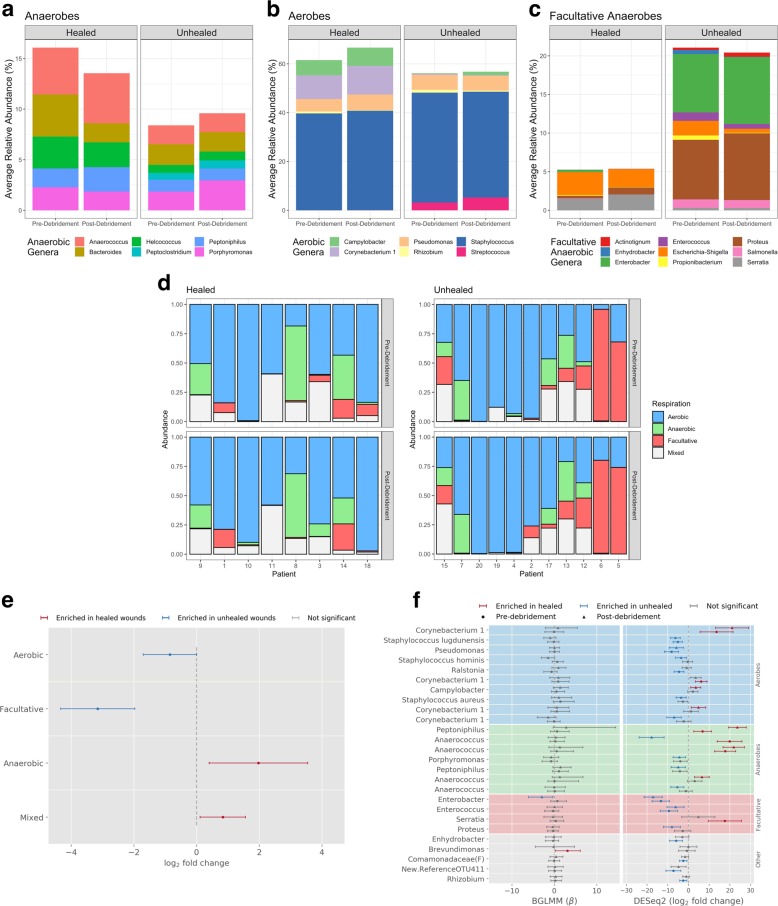
Comparison of healed and nonhealing wounds. Average relative abundance of taxa classified by oxygen requirements (anaerobic (**a**), aerobic (**b**), and facultative anaerobes (**c**)) suggests facultative anaerobes may be predictive of healing outcome. Plots were filtered to show taxa with >0.5% average relative abundance within each sample type and outcome. Cumulative relative abundance of aerobes, anaerobes, facultative anaerobes, and unassigned taxa in wound samples that did or did not heal (**d**). Healed wounds are ordered by estimated wound age when known; unhealed wounds are ordered by treatment time up to the point of medical record data collection. Differential abundance analysis of healing outcomes for taxa with different oxygen requirements using DESeq2 indicated substantial enrichment of facultative anaerobes in nonhealing wounds (**e**). Error bars indicate estimated 95% confidence interval (1.96× standard error, *n* = 19). Taxonomic associations (OTU with average relative abundance > 0.1%) identified by BGLMM or DESeq2 with healed or unhealed wounds, comparing pre-debridement or post-debridement samples from each outcome, indicates significant enrichment of *Enterobacter* in nonhealing wounds (**f**). Error bars indicate 95% confidence intervals for BGLMM inference (*n* = 19) and estimated 95% confidence interval (1.96× standard error, *n* = 19) for DESeq2.

The previous study^7^ also indicated that debridement appeared to decrease the abundance of anaerobes, as assessed 2 weeks post-debridement, in wounds that healed within 12 weeks, but not in wounds that did not heal in that time frame. It was therefore of interest to determine whether this differential response in anaerobes could be seen immediately after debridement. Following grouping into types of oxygen requirement (aerobes, anaerobes, and facultative anaerobes), small but qualitatively similar trends were observed here. In wounds that healed, debridement caused a small decrease in the average relative abundance of anaerobes, from 16.1 ± 24.1% pre-debridement to 13.6 ± 18.8% immediately post-debridement (Fig. 4a), and a small increase in the average relative abundance of aerobes, from 61.5 ± 25.9% pre-debridement to 66.6 ± 21.4% immediately post-debridement (Fig. 4b). Unhealed wounds showed a slight increase in anaerobes and little change in aerobes from debridement (Fig. 4a, b). Debridement also caused little change in the relative abundance of facultative anaerobes (Fig. 4c). The small differences in response to debridement for the different oxygen-requirement types (anaerobes, aerobes, and facultative anaerobes) were not statistically significant for both healed and unhealed wounds (Supplementary Fig. 9), supporting the idea that differences previously observed develop gradually over the days after debridement.

### Differential abundance of facultative anaerobes in healed vs. unhealed wounds

Although debridement did not appear to differentially affect the composition of oxygen-requirement types in healed vs. unhealed wounds among our samples, a large contrast was seen when comparing the relative abundance of facultative anaerobes in healed vs. unhealed wound samples (pre- or post-debridement). In unhealed wounds, the average relative abundance of facultative anaerobes was 20.8 ± 29.7%, compared to 5.32 ± 7.21% in healed wounds (Fig. 4c). This difference was not statistically significant using a two-sided Wilcoxon rank-sum test, indicating that there was no significant qualitative bias of facultative anaerobes in healed vs. unhealed samples. However, this nonparametric test is insensitive to the magnitude of the differences, i.e., heavy enrichment for facultative anaerobes may be associated with poor healing while mild enrichment has little effect. Examination of the data suggested a high variation in the abundance of facultative anaerobes among patients, especially those with unhealed wounds (Fig. 4d). Indeed, the frequency distribution of facultative anaerobes was found to differ significantly between healed and unhealed wounds (Kolmogorov–Smirnov test: *p* = 1.5 × 10^−4^), and the variance in these distributions significantly differed from each other (Bartlett’s test: *p* = 1.0 × 10^−6^; Fligner–Killeen test: *p* = 0.01). We therefore analyzed the differential abundance of taxa having different oxygen requirements using DESeq2’s variance-stabilizing transformation, which can better account for heteroscedasticity of abundance across samples. Using DESeq2, we found that aerobes and especially facultative anaerobes are significantly more abundant in wounds that did not heal, while anaerobes are more abundant in wounds that did heal (Fig. 4e).

To identify which specific OTUs were associated with healing outcomes, we applied DESeq2 and BGLMM method to compare the wound (pre- and post-debridement) samples of healed vs. unhealed wounds. Overall, most associations of individual OTUs with healing status were not consistently identified by both methods, likely due to the small sample size and diversity of wound colonization among patients. One result found by both methods is that *Enterobacter*, a facultative anaerobe, is associated with nonhealing (Fig. 4f). We also applied DESeq2 to identify skin OTUs associated with healing outcomes. Only one OTU, *Corynebacterium*, was significantly associated with healed wounds; none were associated with unhealed wounds (Supplementary Fig. 10).

## Discussion

We enrolled 20 patients with chronic wounds to characterize the microbial composition of the wound surface exposed by a single, sharp debridement event and assess whether microbial taxa could be predictive of clinical outcomes. While outcomes are certainly influenced by multiple host factors and other clinical factors, we focused on whether microbial taxa were associated with outcomes. Taxonomy summaries and diversity metrics of skin and wound microbiomes were consistent with previous reports^5–7,36^. We applied a novel Bayesian generalized linear mixed model, in addition to DESeq2, to statistically assess associations of individual OTUs with specific sample types (Supplementary Note 3).

BGLMM and DESeq2 agreed on the identification of several taxa that were strongly enriched in wound samples compared to skin. In line with prior assessments of skin and chronic wounds^5–7,36^, the common skin commensals *Micrococcus*, *Paracoccus*, and *Kocuria* were significantly associated with skin using both methods, and DESeq2 further identified a number of *Corynebacterium* and *Staphylococcus* OTUs (*S. hominis*, *S. haemolyticus*, and *S. cohnii*) associated with skin. The known pathogens and wound colonizers *S. aureus*, *S. capitis*, *Proteus*, *Enterobacter*, *Helcococcus*, and *Pseudomonas* were significantly associated with wounds by both methods. Notably, *Staphylococcus* OTUs were associated with both skin and wounds, and species-level associations were only resolved after reannotation of those OTUs using tools outside of the standard QIIME pipeline and SILVA128 database. This drawback highlights the utility of higher resolution 16S analysis methods and annotation, such as “amplicon sequence variant” approaches^37^, or shotgun metagenomics. This has been demonstrated in a recent study showing strain- and species-specific effects in the wound microbiome^7^. Nonetheless, the agreement between DESeq2 and BGLMM on these results increases confidence in the associations identified here, and may prompt further testing of associations found by only one method.

The practice of wound debridement is based on expected impacts on both host physiology and wound microbiota. We swabbed wound surfaces before and then immediately after a single, sharp debridement event in an outpatient clinic. No significant difference in the microbiome composition was detected, either in abundance of OTUs or in abundance of taxa grouped by oxygen requirement (aerobe, anaerobe, facultative anaerobe, and mixed/other/NA). Therefore, we infer that the prior finding of anaerobe depletion at 2 weeks post-debridement results from a gradual shift over days. It should be noted that the small size of this study limits its power to detect small community changes. In addition, Levine’s technique can sample exudate from deep tissue^38^, so swabbing itself may disguise small differences in the wound before and after debridement. Nevertheless, the finding that the composition of the wound surface microbiome immediately exposed by sharp debridement is not significantly different from the pre-debridement wound suggests that the roles of host-associated factors, such as moderation of inflammation, as well as total microbial bioburden, warrant further study. Furthermore, these findings support the principle that debridement should be utilized frequently and aggressively to be most effective^39^.

Wounds were followed up to ~6 months after sampling, enabling patients to be grouped by healing vs. nonhealing outcome after 6 months. In this study, the outcome reflects time since sampling rather than true wound age. The “healed” outcome therefore includes both wounds with age <6 months as well as those with age >6 months that originated before the time of sample collection and healed before patient data were collected. “Unhealed” wounds all had age >6 months. While 12 weeks after initial presentation has been used in other microbiome studies^6,7^ as the assessment time point to distinguish healed from unhealed wounds, 12 weeks was not a practical distinction in our study, in which few wounds healed within that time frame.

When comparing the microbiomes (pre-debridement) of healed vs. unhealed wounds, a notable finding was the over-representation of facultative anaerobes as a group in the microbiome of nonhealing wounds. In contrast, healed wounds appeared to be enriched for anaerobes. It is tempting to speculate that infections in which strict anaerobes play a key role are more easily cleared as the wound heals and the oxygen level increases in the tissue^40^, disfavoring anaerobic organisms. On the other hand, infections in which facultative anaerobes play a key role, however, would be more tolerant to the changing conditions of a healing wound and may thus persist. This interpretation has implications for our understanding of treatments based on increasing the oxygen tension in the wound (e.g., hyperbaric oxygen^41^), for which conflicting literature exists with regard to efficacy^30^. In particular, the presence of pathogenic facultative anaerobes may render the wound refractory to oxygen therapies, suggesting that oxygen therapies should be targeted against wounds with low levels of facultative anaerobes. Another intriguing possiility is that facultative anaerobes may better tolerate the substantial oxygen gradients within the biofilm itself, causing persistence of the biofilm, as recent studies indicate that variable oxygen tension is a dominant stress in the high-density environment of the biofilm^42,43^. In that case, the association of facultative anaerobes with nonhealing would be a consequence of the selective environment within the biofilm. Alternatively, the association of facultative anaerobes with nonhealing may reflect a correlation to a different feature that causes poor healing. For example, the facultative anaerobe metabolism may be an incidental trait in organisms that are particularly problematic in chronic wounds for other reasons. Aside from the mechanism, higher levels of facultative anaerobes may still be useful as prognostic markers of more resilient communities that inhibit healing. Further experimental studies would be needed to probe the influence of facultative anaerobes suggested here.

Taxonomic associations with wound healing are of special interest, as they may point toward species that, in combination with other host-related and clinical factors, promote or delay healing or which may act as biomarkers of healing status. The analyses of individual taxa by both DESeq2 and BGLMM revealed that the genus *Enterobacter*, a facultative anaerobe, was associated with non-healing status. In addition, we did not observe *Enterobacter* in normal skin from the patients in this study, i.e., *Enterobacter* was exclusive to wounds. While *Enterobacter* has been reported in chronic wounds^9,12^, its potential role has been understudied relative to the more abundant *Staphylococcus* and *Pseudomonas* taxa. Interestingly, *Enterobacter* species are a common causative organism of acute infections; in one study, *Enterobacter* was found to be a negative prognostic indicator for surgical site infections developed after neurosurgery^44^. We suggest that further attention to the possible role of *Enterobacter* in chronic wounds is warranted.

Other taxonomic associations with healing outcomes were detected by only one method, likely due to the small sample size and natural heterogeneity of wounds among patients. BGLMM analysis indicated that healed wounds were positively associated with *Brevundimonas*. DESeq2 appeared to be more sensitive to associations in general, with healed wounds positively associated with *Anaerococcus*, *Peptoniphilus*, *Corynebacterium*, and *Serratia*. DESeq2 also identified healing to be negatively associated with *Enterococcus*, *Staphylococcus*, *Pseudomonas*, and *Proteus*. The facultative anaerobe *Proteus* is an intriguing candidate, because, like *Enterobacter*, its overall abundance in nonhealing wounds is relatively high (e.g., Supplementary Fig. 8). In addition, a single *Corynebacterium* OTU from skin samples was positively associated with healing. The less robust findings represent hypotheses that may be further tested in larger studies. The results also validate BGLMM as a novel statistical model complementary to DESeq2 for future association studies.

This work is constrained by several limitations. The power of this study to identify correlations (or associations) was limited by its small cohort size (20 patients), such that only associations of relatively large effect would be detected. Larger cohorts will be important to test the findings, particularly correlations discovered by the only one statistical method. Within patients, sampling was limited to one swab pre- and one swab post-debridement per wound, both due to the possibility of small wound sizes and concerns raised in discussion with the Institutional Review Board regarding repeated sampling. While limited sampling is a potential concern, the concordance between pre- and post-debridement samples found here suggests that swabbing itself is not a major source of variation. On the other hand, while all wounds underwent sharp debridement, the instrument used for debridement and degree of debridement were not controlled and may contribute to patient-to-patient variability in the results.

For the majority of OTUs, while we sequenced the V1–V3 loops of the 16S rRNA gene, taxonomic resolution was limited to the genus level. This is likely due to the OTU clustering threshold (97% identity) and reference database (SILVA128). Additional analysis of OTUs of interest (*Staphylococcus*) allowed tentative species-level annotation, although metagenomic sequencing would enable more confident assignments.

Finally, the microbiome data collected here are compositional, i.e., absolute amounts of bacteria are unknown. While we know that negative control samples yielded undetectable amounts of DNA and nearly all skin and wound samples (59/60) yielded detectable amounts^35^, quantitative information on bacterial loads would be useful for interpreting the detection of species (e.g., contamination from reagents) in the negative controls. In addition, bacterial load is likely to be affected by debridement, and correlation analysis might yield more physiologically relevant associations given absolute quantitative data. While swabbing may not be appropriate for absolute quantitation, microbiome sampling by other means (e.g., biopsy^25^) might be more amenable. However, the relevance of quantitative microbiological data for clinical outcomes, and the techniques for collecting these data, are not yet clear^23^.

In summary, the results of this study show that sharp debridement does not have a large immediate impact on the composition of wound microbiota. In addition, the study identified the abundance of facultative anaerobes in toto as a negative prognostic factor for healing in chronic wounds, and the facultative anaerobe *Enterobacter* was specifically associated with nonhealing vs. healing wounds. Understanding the mechanism of these associations will require causal inference (e.g., from time series data) and/or experimental models. Further work in these directions may be fruitful for understanding the contribution of the microbiome to wound healing as well as personalizing therapeutic recommendations based on wound-specific microbiomes.

## Methods

### Ethics statement

Clinical sample collection was performed at Ridley–Tree Center for Wound Management at Goleta Valley Cottage Hospital in accordance with protocols approved by the Cottage Health Institutional Review Board (Study Protocol 17–48u). We recruited a cohort of 20 wound care patients over the course of a week and a half, and collected samples after obtaining informed, written consent from the patient.

### Collection of clinical samples

Four clinically classified chronic wound types were sampled (diabetic ulcers, venous wounds, arterial wounds, and pressure ulcers), with five patients per wound type. Exclusion criteria were as follows: patients under the age of 18, in the intensive care unit, or presenting with an unrelated non-wound infection. All patients underwent sharp debridement, but the extent and depth of debridement, as well as the type of instrument (curette, scalpel, scissors, or tissue nipper), was not standardized and was determined by the treating physician (Table 1). Debridement was not conservative and was undertaken until bleeding was observed. Sterile Copan FLOQSwabs 520C were pre-wetted with sterile PBS prior to all sample collections. During a single patient visit, wound swabs were collected pre-debridement and 1–2 min post-debridement, and a healthy skin swab was collected from the contralateral limb. Wound samples were collected from the area of debridement. All skin and wound samples were collected by employing Levine’s technique; gentle pressure was applied as the swab was wiped and rolled across a ~1 cm^2^ area of healthy granulation tissue for approximately 30 s. Clinical swabs were placed back into the dry, sterile collection tube and stored at 4 °C for no more than 4 h before being processed. Negative control samples from the wound center were collected by exposing swabs to air in the collection room for the same duration as wound and skin swab collection. Processing control samples were obtained by exposing swabs to air and reagents in the processing lab analogously to clinical samples. A cell-based microbial mock community (Zymo) was included as a positive control.

### Sample processing and DNA extraction

Samples were transported to UCSB for processing. Swabs tips were broken off into sterile 1.5 mL microcentrifuge tubes, and samples were resuspended in 500 µL sterile 1× TE by vortexing for 2 min at high speed on a multitube vortex adapter. Cells and cell debris were pelleted by centrifugation at 16,000×*g* for 2 min. Totally, 250 µL of supernatant was transferred to a sterile microcentrifuge tube for virus-like particle enrichment and DNA extraction, while the remaining solution and swab tip were retained for whole-microbiome DNA extraction. All subsequent purification and extraction steps were performed as described in^35^. Briefly, bacterial DNA was extracted by high activity lysozyme treatment, proteinase K digestion, chemical lysis, bead beating, and final DNA purification with a PureLink Genomic Mini Kit, with all samples eluted into 25 µL of 1× TE. Extracted DNA was quantified using a Qubit 3.0 instrument, dsDNA HS kit, and 5 µL of sample. Of the 20 skin samples, only one sample was below the limit of detection (40 pg); the minimum total DNA detected was 1 ng, maximum was 17.3 ng, and average was 3.0 ng. All wound samples produced sufficient DNA for Qubit quantification; the minimum total DNA detected was 48.3 ng, maximum was 11.5 µg, and average was 2.92 µg. All negative controls were below the limit of detection, and positive controls yielded sufficient DNA for sequencing.

### Library preparation and sequencing

16S rRNA sequencing libraries were generated by two-step polymerase chain reaction (PCR) for each sample. In the first step, V1–V3 loops were amplified using custom adapter primers composed of universal 16S primers “27F” and “534R” and Illumina Nextera indexing adapter sequences. Adapter PCR was done in 25 µL reactions containing 11.5 µL of template, 0.5 µL of each primer at 10 µM, and 12.5 µL of KAPA HiFi HotStart ReadyMix. Adapter PCR was performed under the following conditions: denaturation/activation at 95 °C for 3 min, followed by 25 cycles of denaturation at 95 °C for 30 s, annealing at 55 °C for 30 s, and extension at 72 °C for 30 s. PCR products were purified with 20 µL AMPureXP beads and eluted into 50 µL of 10 mM Tris pH 8.5. In the second step, Illumina Nextera XT indices were added by PCR in 50 µL reactions containing 5 µL of product from adapter PCR, 5 µL of Index 1, 5 µL of Index 2, 25 µL of KAPA HiFi HotStart ReadyMix, and 10 µL of water. Eight cycles of PCR were conducted under the same conditions as adapter PCR. Indexed samples were purified with 56 µL of AMPureXP beads, eluted into 25 µL of 10 mM Tris pH 8.5, quantified with a Qubit dsDNA HS kit, normalized and pooled for multiplexing. All samples, including controls, were sufficiently amplified for sequencing. Final library QC was done using an Agilent TapeStation dsDNA 1000 bp kit. The final libraries were sequenced in a single run on an Illumina MiSeq with PE300 V3 chemistry at UCSB’s Biological Nanostructures Laboratory (BNL) sequencing core.

### 16S rRNA bioinformatic analyses

Paired-end reads were uploaded to the QIIME AWS AMI (AMI ID: ami-1918ff72, “qiime-191”)^33^. Initial quality analysis was performed with FastQC. Reads were quality controlled by trimming and quality filtering with trimmomatic using default settings^45^. Read joining was performed with QIIME’s joining script (join_paired_ends.py), using the fastq-join algorithm with default settings. Joined reads were fed into the open OTU picking pipeline (pick_open_reference_otus.py) using default settings. Taxonomy was assigned using the SILVA128 16S reference database clustered at the 97% identity threshold^34^. Representative sequences that could not be aligned using pyNAST were putative human contamination^46^ and excluded from the final BIOM table^47^. The final BIOM table (without PyNAST alignment failures) contained 69 samples (60 experimental + 9 controls), with 22,753 OTUs representing 5,931,472 total counts (median = 65,588). For 608 OTUs annotated as *Staphylococcus* at the genus level, species level annotations were generated using blastn^48^. Representative sequences of each OTU were queried against the NCBI nr/nt nucleotide collection, subset to the Bacillus/Staphylococcus group (taxid: 1385). For each OTU, blast results were parsed by sorting the hit table, in descending order, by bit-score, then *e*-value, and the highest hit for each OTU was retained. The Entrez Direct command line tool was used to obtain the taxonomy lineage corresponding to each accession number from the parsed hit table, and the species name was appended to the OTU table in R.

For differential abundance analyses, the OTU table was filtered to keep only OTUs with least 10 reads in at least 5 samples (filtered table contained 462 OTUs). Three experimental samples contained less than 1000 counts; analyses with the samples excluded yielded similar results to analysis of the full data set, so they are included in the analyses described here. The skin sample from patient 16 contained an abnormally high number of OTUs, and its rarefaction curve did not saturate; this sample, and its associated wound samples, were removed from analysis (Supplementary Fig. 1). Results from DESeq2 and BGLMM were compared with and without patient 16 (Supplementary Fig. 11) to confirm the robustness of taxonomic association analysis to the removal of patient 16 data.

### Diversity and differential abundance analyses

All diversity analyses were performed in RStudio. Briefly, the QIIME BIOM table, tree file, and mapping file were imported and converted to a Phyloseq object^49^. Data were preprocessed to root the tree, reformat the taxonomy levels and strings, add species level annotations for *Staphylococcus* OTUs, transform counts to relative abundance values, filter out controls and insufficiently sequenced samples, and pregenerate genus-agglomerated tables for downstream analyses. Taxonomic summaries (dotplots, barcharts, and boxplots) were generated using the genus-agglomerated tables, with filters indicated in the figure captions. Alpha diversity plots were generated using raw, unfiltered, unrarefied tables. Beta diversity distances were calculated using a genus-agglomerated table with counts normalized by relative abundance, and filtered to retain OTUs with average relative abundance greater than 0.01%. Differential abundance analyses were conducted using the DESeq2 package with the Wald test and parametric fitting^31^. Log_2_(fold-change)s and adjusted *p* values were extracted for each comparison using the contrast function. OTUs with adjusted *p* values less than 0.05 were considered as significantly different in a differential comparison. 95% Confidence intervals (CI) were estimated by 1.96× standard error (lfcSE) reported by DESeq2 package. Note that OTUs whose 95% CI does not include zero might not be significant by the standard of adjusted *p* value, as *p* values were further adjusted by the Benjamini–Hochberg correction for false discovery rate using DESeq2.

Additional packages utilized for analysis and figure generation in RStudio include: genefilter, ggplot2^50^, ggpubr, reshape2, RColorBrewer, viridis, wesanderson, grid, gridExtra, plotly, scales, dplyr, magrittr, data.table, and ape. Additional packages in python include numpy, pandas, scipy, matplotlib, and biom-python.

### Bayesian generalized linear mixed model

To further assess the association of OTU abundances with different sample types (pre-debridement, post-debridement, or skin) and outcomes (healed or unhealed), we modified the model in ref. ^32^ to analyze our data. Let $Y_{ikj}$ denote the detected count of OTU *j* in sample *k* from patient *i* in the sequencing, $i = 1, \ldots ,n$, $k = 1, \ldots ,K$ and $j = 1, \ldots ,J$. In our dataset, three samples were taken from each of the patients. We have *n* = 19 or 20, depending on inclusion or exclusion of patient 16, $K = 3$, and *J* = 462 (OTU table filtered for counts >10 in >5 samples). We assume a negative binomial distribution for $Y_{ikj}$, $Y_{ikj} \sim NB(\mu _{ijk},\,s_j)$, where $\mu _{ikj}$ is the mean of $Y_{ikj}$ and $s_j$ is the overdispersion parameter. $s_j$ accounts for overdispersion of counts $Y_{ikj}$ and we have $Var[Y_{ikj}] = \mu _{ikj} + s_j \times \mu _{ikj}^2$. We use the normal skin sample as the reference group and consider a log linear model for $\mu _{ikj}$, $log(\mu _{ikj}) = r_{ik} + \alpha _j + u_{ij} + \beta _{j1}x_{ik1} + \beta _{j2}x_{ik2}$, where $x_{ik} = (x_{ik1},\,x_{ik2})$ is a vector of covariates. In our study, we use two binary indicators to represent different sample types (i.e., $x_{ik} = (0,\,0)$, $(1,\,0)$, and $(0,\,1)$) mean the skin, pre-debridement, and post-debridement samples from patient $i$, respectively). Regression coefficients $\beta _{j1}$ and $\beta _{j2}$ are parameters that infer the effects of pre- and post-debridement states on the abundance of OTU $j$ relative to its abundance on normal skin, respectively. The difference in the coefficients $\beta _{j2} - \beta _{j1}$ can be used to infer the effect of debridement on OTU $j$. Here, $r_{ik}$ is the normalizing factor for sample $k$ of patient $i$. It accounts for different total counts in samples (e.g., sampling and sequencing depths); $\alpha _j$ is the baseline abundance of OTU $j$ that explains variability in the baseline OTU abundances; $u_{ij}$ is a random effect of OTU $j$ in patient $i$ that accommodates heterogeneity between patients. Since $u_{ij}$ is common for all samples taken from patient $i$, it also induces dependence between abundances of OTU $j$ in the samples of patient $i$. In Bayesian models, unknown parameters are random and specification of a priori distributions for the random parameters (called prior distributions) is required. Bayesian models then update the distributions of the random parameters using observed data and yield a posteriori distributions (called posterior distributions). We assume prior distributions on our unknown parameters such as $r_{ik}$, $\alpha _j$, and $\beta _{jp}$, similar to those in Lee and Sison-Mangus^32^. In particular, we assume independent Laplace distributions for $\beta _{jp}$ (*p* = 1, 2), resulting in Bayesian lasso. The Laplace prior is a sparse inducing prior that improves estimation of $\beta _{jp}$ and facilitates variable selection. We used 95% posterior credible intervals of $\beta _{jp}$, $p = 1,\,2$, and $\beta _{j2} - \beta _{j1}$ to identify significant associations for individual OTUs. We also assume Laplace distributions for $u_{ij}$. For details of the prior specification, see Lee and Sison-Mangus^32^. Since the posterior distribution is not in a closed form, we used a Markov chain Monte Carlo method consisting of Gibbs and Metropolis steps to evaluate the posterior distribution. Median and 95% credible intervals for all $\beta _{jp}$ were reported.

We performed an additional analysis to assess the association of OTU abundances with different samples types in healed and unhealed wounds. We considered pre- and post-debridement samples only and used the BGLMM with two covariates, pre- and post-debridement, and healed and unhealed wounds. The covariates form four different groups: pre-debridement/unhealed, post-debridement/unhealed, pre-debridement/healed, and pre-debridement/unhealed groups. We used the pre-debridement/unhealed group as the baseline and then let ${\rm{log}}(\mu _{ikj}) = r_{ik} + \alpha _j + u_{ij} + \beta _{j1}x_{ik1} + \beta _{j2}x_{ik2} + \beta _{j3}x_{ik3}$ similar to the previous analysis. Here, $\beta _{j1}$, $\beta _{j2}$, and $\beta _{j3}$ represent the effects of the remaining three groups on the abundance of OTU j relative to that of the baseline group. Difference between a pair of $\beta _{jp}$ $\left( {p = 1,2,3} \right)$ can be used to infer difference of OTU abundances between groups.

For all inference using BGLMM, regression coefficient ($\beta _{jp}$) whose 95% credible interval does not include zero were considered to have a statistically significant effect. We conducted inference on the filtered table (462 OTUs) with or without patient 16 for DESeq2 and BGLMM.

## Supplementary information

supplementary-materials

reporting-summary

## Data Availability

All data used to perform the analyses and generate the corresponding figures are publicly available at the Dryad Digital Repository (10.25349/D9TS32). Raw reads are available at NCBI’s Sequence Read Archive (BioProject Accession PRJNA612439).

## Code availability

All code used to perform the analyses and generate the corresponding figures are publicly available at the Dryad Digital Repository (10.25349/D9TS32).
