# Supplementary material for: Microbial predictors of healing and short-term effect of debridement on the microbiome of chronic wounds
Source: NPJ Biofilms Microbiomes. 2020 May 1;6:21. doi: 10.1038/s41522-020-0130-5 (PMC7195478; doi:10.1038/s41522-020-0130-5)
Supplement: Supplementary file 1 — supplementary-materials [file 41522_2020_130_MOESM1_ESM.pdf]

Supplementary Information: Verbanic et al.

Microbial predictors of healing and short-term effect of debridement on the microbiome of chronic wounds

Supplementary Table 1
Supplementary Figures 1-13
Supplementary Notes 1-3

Supplementary Table 1. OTUs found in negative control samples. A table of genus-agglomerated OTUs found in the wound care center negative control samples and the Chen Lab processing negative control samples, and their associated taxonomy. OTUs shown had average relative abundance > 0.1%. Absolute abundances are not known.

OTU_ID	Domain	Phylum	Class	Order	Family	Genus
CP001628.1564668.1566028	Bacteria	Actinobacteria	Actinobacteria	Micrococcales	Micrococcaceae	Micrococcus
GQ258634.1.1318	Bacteria	Proteobacteria	Gammaproteobacteria	Pseudomonadales	Pseudomonadaceae	Pseudomonas
GALZ01007484.194.1638	Bacteria	Cyanobacteria	Chloroplast	uncultured bacterium	uncultured bacterium	uncultured bacterium
JQ825174.1.1458	Bacteria	Proteobacteria	Alphaproteobacteria	Rhodospirillales	Rhodospirillaceae	Skermanella
New.ReferenceOTU749	Bacteria	Proteobacteria	Alphaproteobacteria	Sphingomonadales	Sphingomonadaceae	NA
EF098188.1.1345	Bacteria	Proteobacteria	Alphaproteobacteria	Sphingomonadales	Sphingomonadaceae	Sphingomonas
EU328097.1.1450	Bacteria	Proteobacteria	Alphaproteobacteria	Sphingomonadales	Erythrobacteraceae	Ambiguous_taxa
New.ReferenceOTU1013	Bacteria	Actinobacteria	Actinobacteria	Micrococcales	Micrococcaceae	Kocuria
FJ557790.1.1378	Bacteria	Actinobacteria	Actinobacteria	Corynebacteriales	Corynebacteriaceae	Corynebacterium 1
New.ReferenceOTU967	Bacteria	Actinobacteria	Actinobacteria	Corynebacteriales	NA	NA
New.CleanUp.ReferenceOTU84464	Bacteria	Actinobacteria	Actinobacteria	Micrococcales	Brevibacteriaceae	Brevibacterium
KU515422.1.1474	Bacteria	Bacteroidetes	Cytophagia	Cytophagales	Cytophagaceae	Hymenobacter
New.CleanUp.ReferenceOTU60425	Bacteria	Bacteroidetes	Flavobacteriia	Flavobacteriales	Flavobacteriaceae	Chryseobacterium
New.CleanUp.ReferenceOTU42685	Bacteria	Chloroflexi	Gitt-GS-136	uncultured bacterium	uncultured bacterium	uncultured bacterium
New.CleanUp.ReferenceOTU25360	Bacteria	Proteobacteria	Alphaproteobacteria	Rhodospirillales	Rhodospirillaceae	uncultured
GDGZ01024135.295.1812	Bacteria	Proteobacteria	Gammaproteobacteria	Xanthomonadales	Xanthomonadaceae	Stenotrophomonas
AJ310688.1.1503	Bacteria	Proteobacteria	Gammaproteobacteria	Vibrionales	Vibrionaceae	Vibrio
KF841934.1.1385	Bacteria	Proteobacteria	Gammaproteobacteria	Enterobacteriales	Enterobacteriaceae	Klebsiella
GQ491333.1.1396	Bacteria	Proteobacteria	Gammaproteobacteria	Enterobacteriales	Enterobacteriaceae	Escherichia-Shigella
LECC01000011.1.1290	Bacteria	Proteobacteria	Gammaproteobacteria	Enterobacteriales	Enterobacteriaceae	Salmonella
New.ReferenceOTU180	Bacteria	Proteobacteria	Gammaproteobacteria	Enterobacteriales	Enterobacteriaceae	NA
JF830179.1.1509	Bacteria	Proteobacteria	Gammaproteobacteria	Pseudomonadales	Moraxellaceae	Acinetobacter
New.ReferenceOTU781	Bacteria	Proteobacteria	Gammaproteobacteria	Oceanospirillales	Halomonadaceae	Halomonas
GU731299.1.1390	Bacteria	Proteobacteria	Betaproteobacteria	Burkholderiales	Comamonadaceae	NA
New.CleanUp.ReferenceOTU10576	Bacteria	Proteobacteria	Betaproteobacteria	Burkholderiales	Burkholderiaceae	Ralstonia
New.CleanUp.ReferenceOTU107792	Bacteria	Proteobacteria	Betaproteobacteria	Burkholderiales	Oxalobacteraceae	Massilia
KC286845.1.1502	Bacteria	Proteobacteria	Betaproteobacteria	Burkholderiales	Alcaligenaceae	Ambiguous_taxa
ACOR01000003.1451.2935	Bacteria	Proteobacteria	Alphaproteobacteria	Rhizobiales	Brucellaceae	NA
New.ReferenceOTU643	Bacteria	Proteobacteria	Alphaproteobacteria	Rhizobiales	Phyllobacteriaceae	Mesorhizobium
GQ360006.1.1437	Bacteria	Proteobacteria	Alphaproteobacteria	Rhizobiales	Rhizobiaceae	Rhizobium
AAAA02020712.626.2096	Bacteria	Proteobacteria	Alphaproteobacteria	Rhizobiales	Methylobacteriaceae	Methylobacterium
LSJI01000180.148730.150191	Bacteria	Proteobacteria	Alphaproteobacteria	Caulobacterales	Caulobacteraceae	Phenylobacterium
EF600592.1.1339	Bacteria	Proteobacteria	Alphaproteobacteria	Caulobacterales	Caulobacteraceae	Brevundimonas
CBVZ010000002.74463.76000	Bacteria	Firmicutes	Bacilli	Bacillales	Listeriaceae	Listeria
FJ957654.1.1486	Bacteria	Firmicutes	Bacilli	Bacillales	Bacillaceae	Bacillus

DQ532350.1.1524	Bacteria	Firmicutes	Bacilli	Bacillales	NA	NA
New.ReferenceOTU85	Bacteria	Firmicutes	Bacilli	Lactobacillales	P5D1-392	Ambiguous_taxa
FR873975.1.1545	Bacteria	Firmicutes	Bacilli	Lactobacillales	Lactobacillaceae	Lactobacillus
JAHK01000002.1485485.1487033	Bacteria	Firmicutes	Bacilli	Lactobacillales	Enterococcaceae	Enterococcus
GBKB01000906.322.1853	Bacteria	Firmicutes	Bacilli	Bacillales	Staphylococcaceae	Staphylococcus
New.ReferenceOTU915	Bacteria	Firmicutes	Bacilli	Lactobacillales	Streptococcaceae	Streptococcus
New.ReferenceOTU1058	Unassigned	NA	NA	NA	NA	NA
FJ957443.1.1492	Bacteria	Actinobacteria	Actinobacteria	Propionibacteriales	Propionibacteriaceae	Propionibacterium
New.ReferenceOTU133	Bacteria	Actinobacteria	Actinobacteria	Micrococcales	Dermabacteraceae	NA
GBFX01069610.194.1692	Bacteria	Actinobacteria	Actinobacteria	Corynebacteriales	Nocardiaceae	Rhodococcus
New.ReferenceOTU131	Bacteria	Actinobacteria	Actinobacteria	Propionibacteriales	Nocardioidaceae	Nocardioides
DQ298283.1.1371	Bacteria	Actinobacteria	Actinobacteria	Propionibacteriales	Nocardioidaceae	NA
New.CleanUp.ReferenceOTU79675	Bacteria	Actinobacteria	Actinobacteria	Micrococcales	Microbacteriaceae	NA

Supplementary Figure 1. QIIME pipeline and OTU tables quality control. (a) Percent of reads from each sample passing quality filters in major steps of the QIIME pipeline to prepare the full OTU table. CL1 is the negative control sample collected in the lab. Wound samples have a notably lower percentage of reads passing OTU picking, primarily due to contamination by human DNA. (b) Blastn (2.9.0+) results for representative sequences of OTUs that passed or failed pyNAST alignment (5,000 subsampled OTUs in each case) against NCBI “Human genomic + transcript” and “16S ribosomal RNA sequences (Bacteria and Archaea)” databases. Failed OTUs are primarily human DNA and unmatched sequences to 16S ribosomal RNA database. Number in parentheses indicates the number of OTUs. (c) Distribution of average relative abundance and number of samples in which the OTU was detected, for OTUs in the full OTU table (without pyNAST failures). Bar histograms are shown for average relative abundance and number of samples. (d) Depth of sampling visualized by the cumulative relative abundance composed of the top *k* most abundant OTUs in each sample. A sample from patient 16 showed insufficient sequencing depth and was excluded from downstream analysis. (e) For some analyses (DESeq2 and BGLMM), the full OTU table was further filtered to include only OTUs present in >5 samples with >10 counts per sample. Bar chart shows the relative abundance accounted for by OTUs in the filtered table vs. OTUs only present in the full table.

Supplementary Figure 1

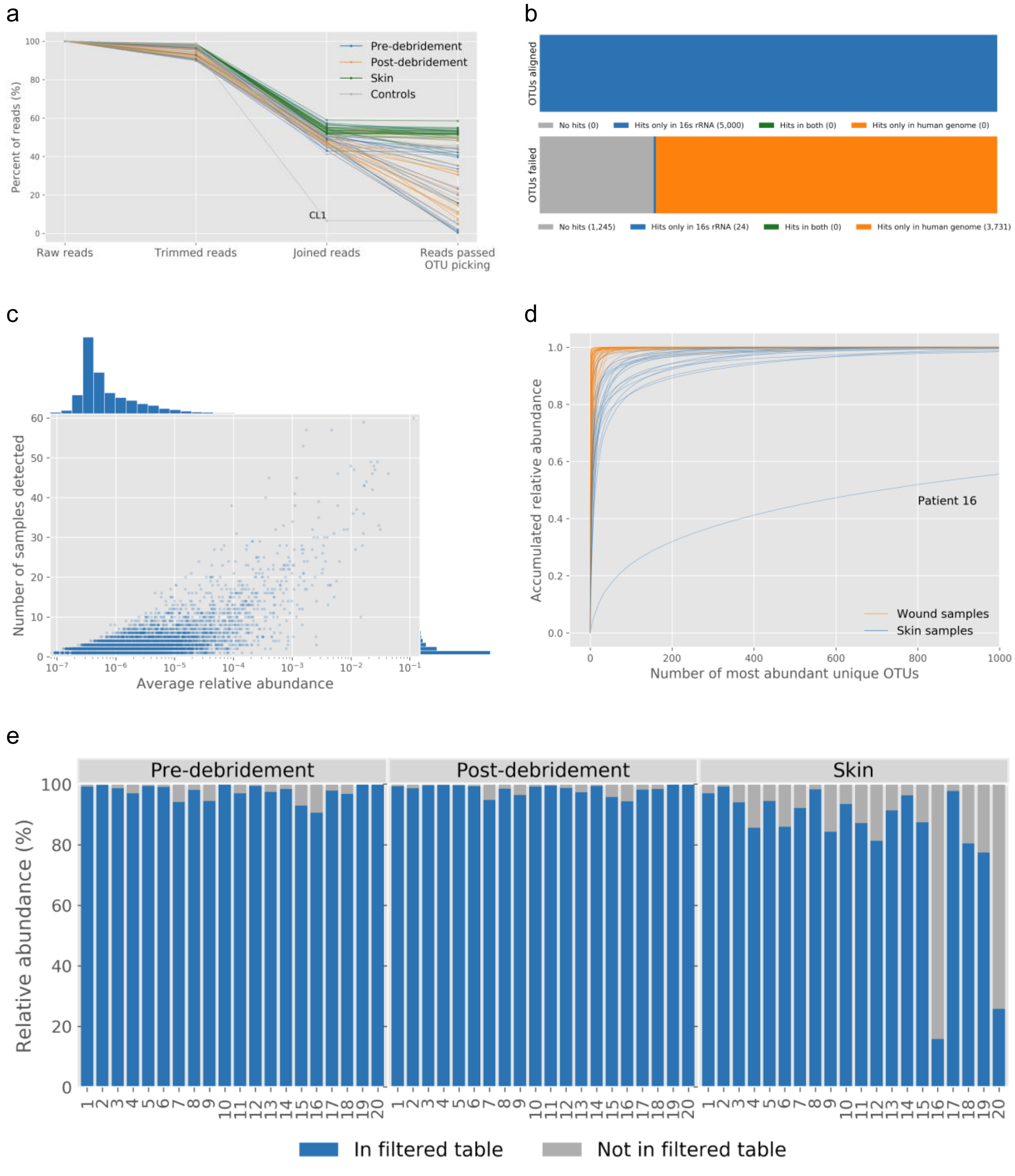

Supplementary Figure 2. 16S rRNA sequencing recapitulates microbial mock communities and previously reported skin & wound microbiota. Expected and observed relative abundances of genera in the microbial mock community positive control (a). Average relative abundance of top 4 phyla across all skin and wound samples (b).

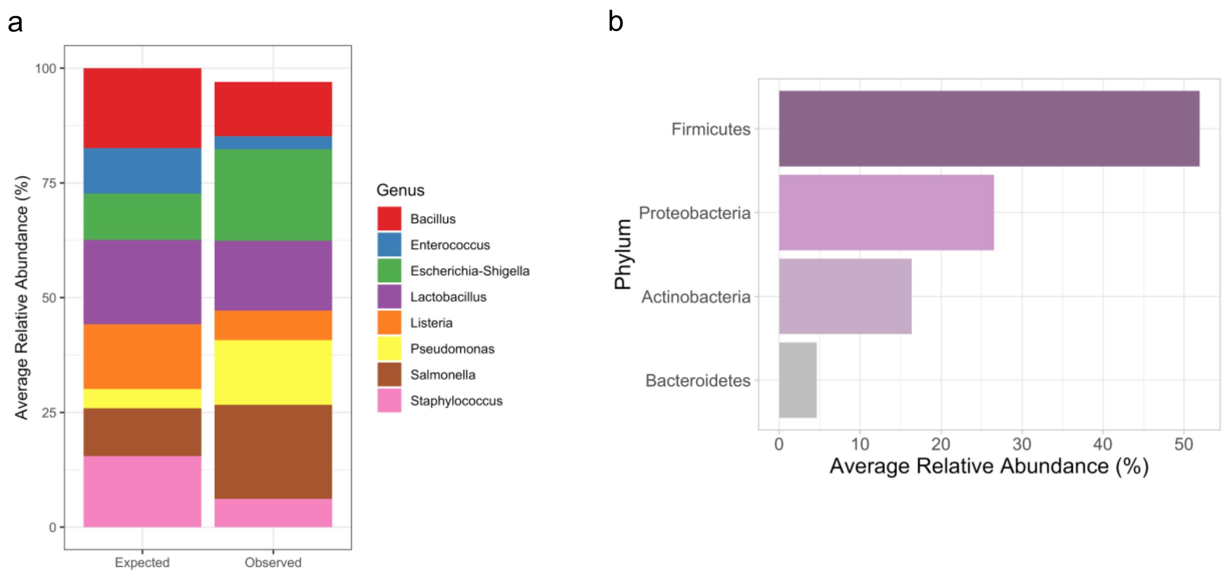

Supplementary Figure 3. Bacterial diversity of skin and chronic wound microbiomes. (a) Boxplots of richness (Chao1) and Shannon indices for each sample type indicate that while pre- and post-debridement wound samples have similar within-sample diversity, skin samples are significantly more diverse. Lower and upper bounds of the box correspond to the first and third quartiles, center line indicates the median, and whiskers extend up to 1.5 x interquartile range; any points beyond the whiskers are outliers. Data points corresponding to a single patient are connected by grey lines. Averages were compared with paired, two-sided Wilcoxon signed-rank tests, resulting in the p-values shown. Error bars shown in the Chao1 plot are standard errors of the richness estimation. (b) Heat map of relative abundance of the 300 most abundant OTUs. As expected based on previous studies [5], the microbiomes of wound samples were less diverse than those of skin samples. Average taxonomic richness of OTUs, as estimated by the Chao1 index [36], was significantly (approximately 10-fold) lower in wound samples than in skin samples). Visual inspection of the distribution of OTU abundances, as well as calculation of diversity by the Shannon index, which accounts for both OTU richness and abundance, indicated that wound samples tended to be dominated by a handful of constituents, while skin communities had a more even distribution of taxonomic abundance. These results are consistent with prior findings in chronic wounds as well as other systems in which pathological states exhibit reduced microbiome diversity [8,37,38].

a b

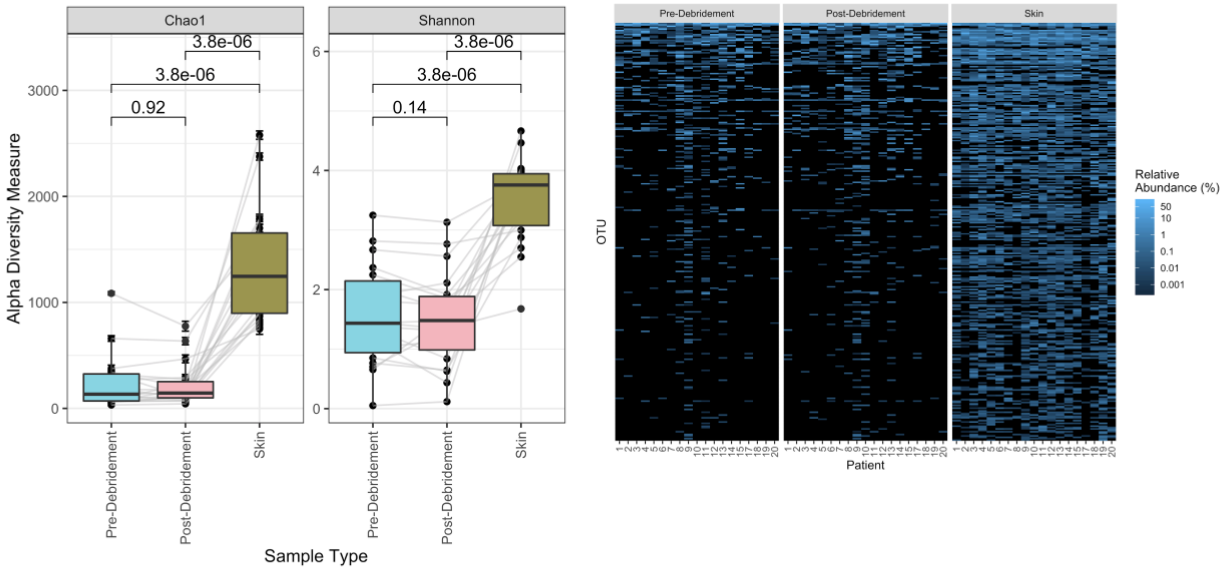

Supplementary Figure 4. Overall comparison of bacterial communities in skin vs. chronic wound microbiomes. Ordination based on the OTU table (normalized by relative abundance and filtered for OTUs with >0.01% relative abundance in >5 samples) using Bray-Curtis dissimilarity (a), unweighted UniFrac (c), and weighted UniFrac (e) indicate that low abundance taxa distinguish skin (yellow cross) from wound samples (pre-debridement: red; post-debridement: aqua). Clinical wound type is indicated by marker shape, and each patient's pre- and post-debridement samples are connected with black lines. Pairwise distances among sample types (pre-debridement, post-debridement, and skin) from the same patient, using Bray-Curtis (b), unweighted UniFrac (d) and weighted UniFrac (f) metrics. For b, d, f, and g, lower and upper bounds of the boxes correspond to the first and third quartiles, center line indicates the median, and whiskers extend up to 1.5 x interquartile range; any points beyond the whiskers are outliers. Averages were compared by Wilcoxon signed-rank tests (p-values shown) and data from each patient are connected by grey lines. Number of OTUs (with average relative abundance >0.1%) found exclusively on skin or wounds, or shared between both, per patient (g), shows that most OTUs are exclusive to skin. However, shared OTUs account for the greatest relative abundance in the wounds of all patients and in the skin of most patients (h).

Supplementary Figure 4

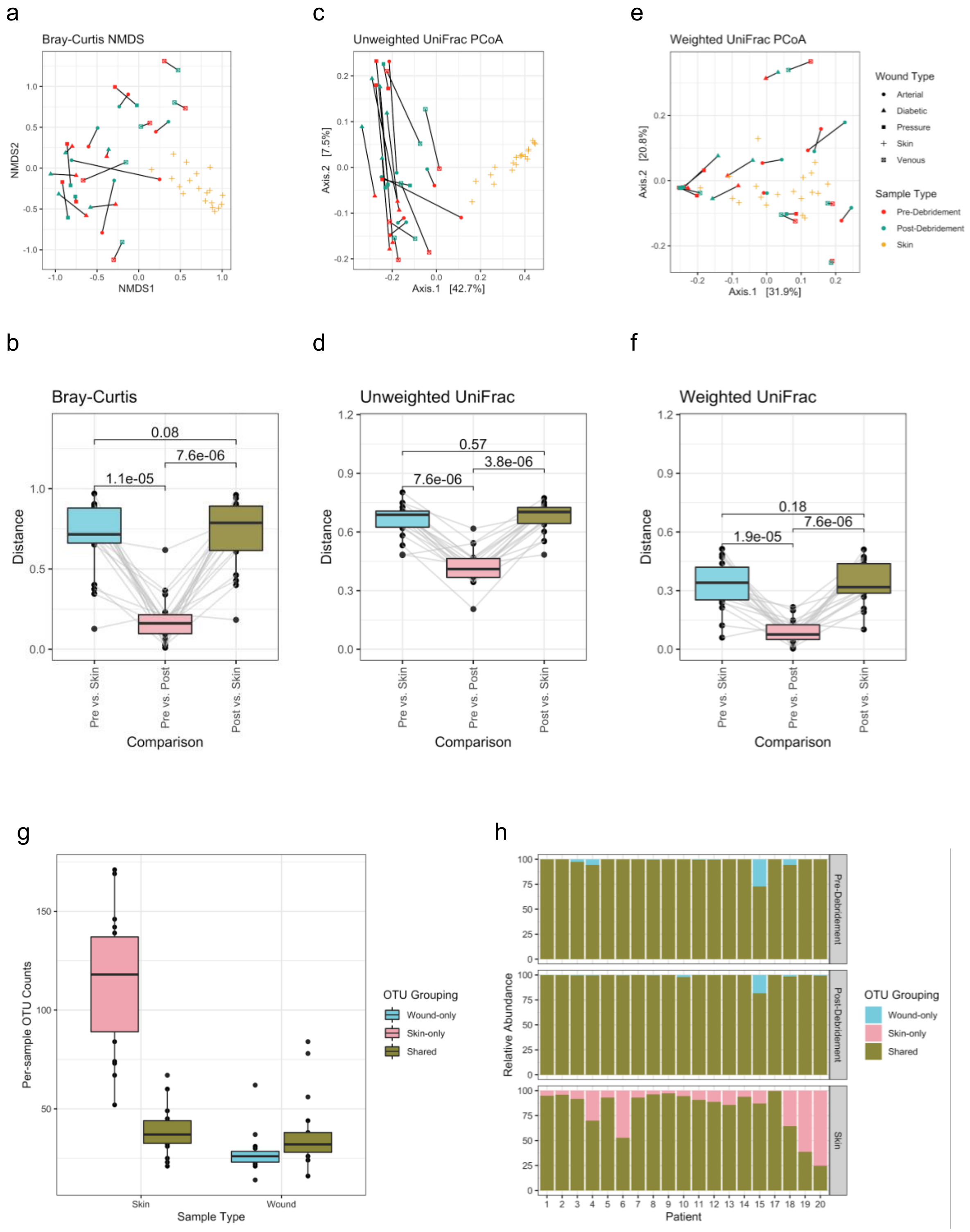

Supplementary Figure 5. Taxonomic summary of skin and wound microbiota. Taxonomic boxplots of genera with >0.1% average relative abundance, for each sample type. Lower and upper bounds of the boxes correspond to the first and third quartiles, center line indicates the median, and whiskers extend up to 1.5 x interquartile range; any points beyond the whiskers are outliers. Of taxa with average relative abundance >1% across all patients, *Proteus, Enterobacter, Campylobacter, Bacteroides,* and *Helcococcus* were found almost exclusively in wounds. On the other hand, skin samples contained several major constituents not found in the wounds, including *Propionibacterium, Enhydrobacter, Micrococcus, Kocuria,* and *Brevundimonas*. Shared taxa also showed differences in abundance between skin and wounds. For example, *Corynebacterium* was present in both skin and wounds, but its average relative abundance was greater on skin. Conversely, *Staphylococcus, Porphyrmonas,* and *Anaerococcus* were found on both skin and wounds, but their average relative abundances were much greater in wounds.

Supplementary Figure 5

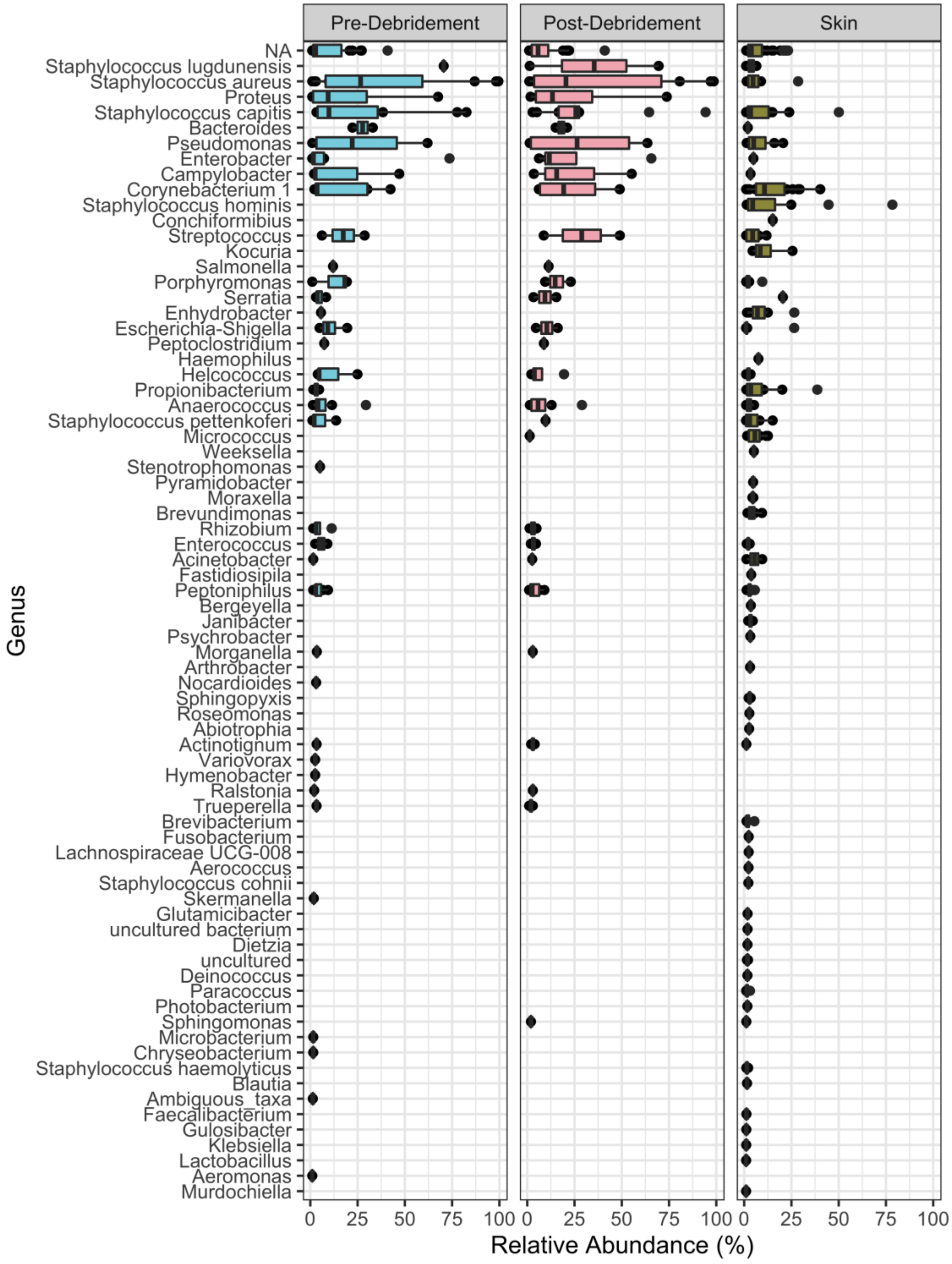

Supplementary Figure 6. BGLMM vs. observed data. Comparison of predicted counts under BGLMM and observed counts in all pre-debridement (a), post-debridement (b), and skin (c) samples (except for patient 16). Pearson's and Spearman's correlation coefficients are shown. Correlations without over-estimated OTUs whose predictions are above 10-fold of true counts (grey dots) were shown in parentheses to illustrate the effects of outliers. Note that Pearson's correlation coefficient is sensitive to outliers and, specifically for skin samples, over-estimated counts led to low Pearson's correlation coefficient. Reasonably robust Spearman's correlation coefficients shows a good correlation of count rank for OTUs.

a b c

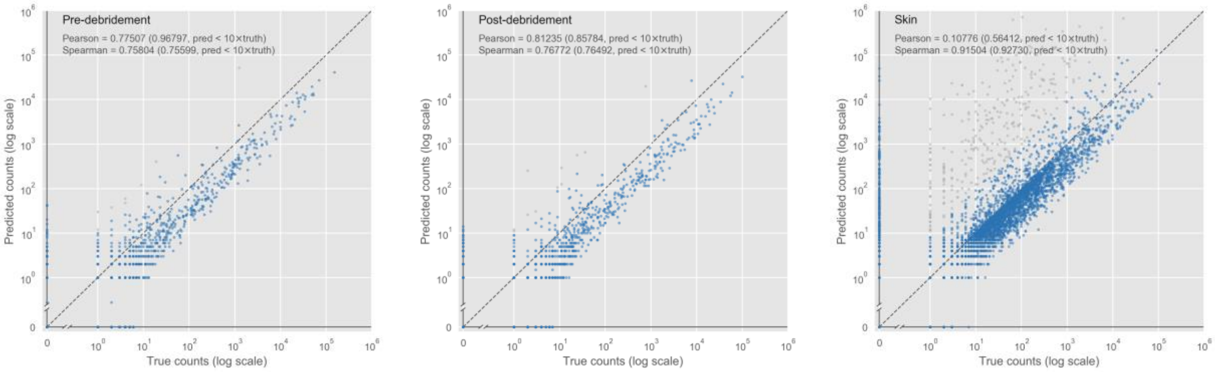

Supplementary Figure 7. Relative abundance of significant OTUs detected using DESeq2 and BGLMM, in pre-debridement vs skin comparison using the filtered OTU table without patient 16. In general, significant OTUs detected using BGLMM constituted a majority of abundance in the wound samples but less abundance in skin samples. On the other hand, significant OTUs detected using DESeq2 had higher relative abundance in skin samples, and composed little abundance (less than 20%) in some wound samples (patients 2, 4, 11, and 18). Overall, OTUs identified as significant by BGLMM represented >20% of the abundance in all wound samples, while OTUs identified as significant by DESeq2 comprised only a small fraction (<20%) in four wound samples; this greater detection of wound-associated OTUs by BGLMM compared to DESeq2 would be consistent with the ability of BGLMM to account for patient-specific variability.

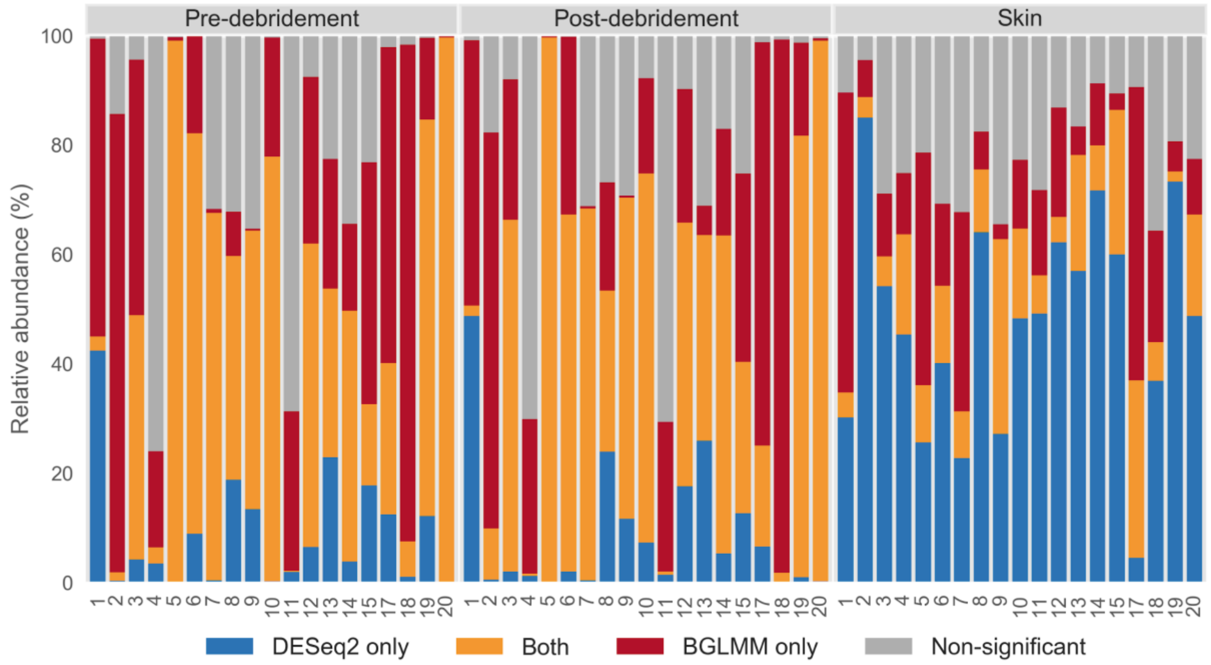

Supplementary Figure 8. Comparison of healed and non-healing wounds. No difference was found between these groups in bacterial diversity measures (a). Within-patient beta diversity measurements (b) show no significant differences in UniFrac distances between samples for healed vs. unhealed wounds. Lower and upper bounds of the boxes correspond to the first and third quartiles, center line indicates the median, and whiskers extend up to 1.5 x interquartile range; any points beyond the whiskers are outliers.

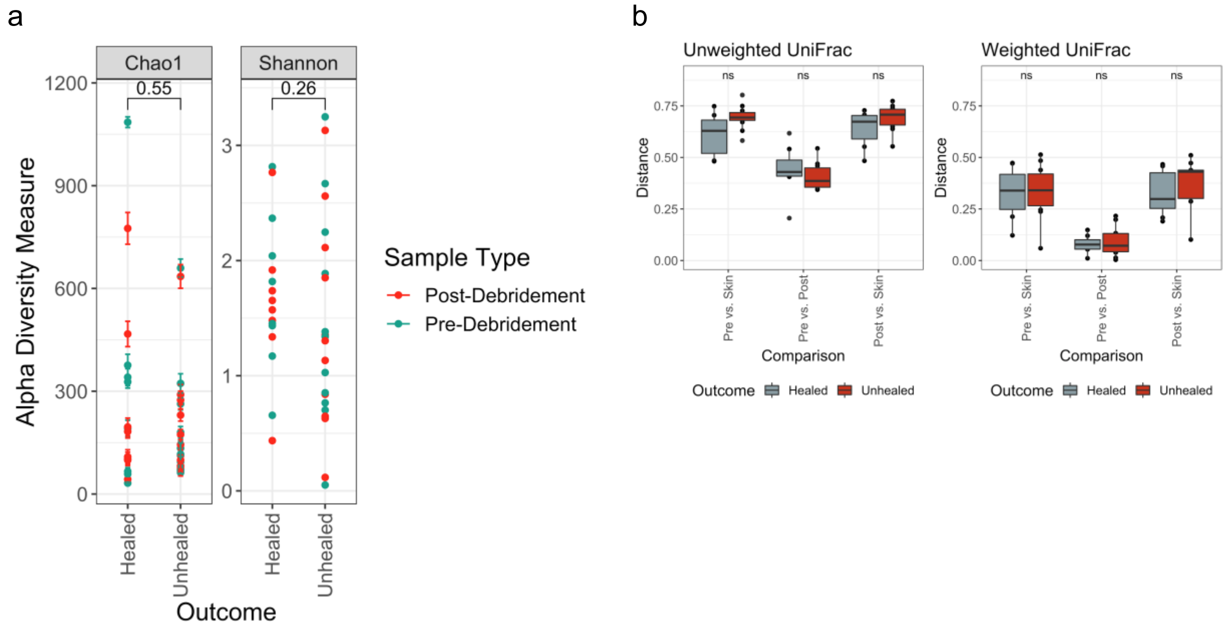

Supplementary Figure 9. Oxygen requirement summaries & statistics. Relative abundance boxplots of anaerobes (a) and aerobes (b) in pre- and post-debridement samples, of healed and unhealed wounds. Pre- and post-debridement samples are compared by paired, two-sided Wilcoxon rank-sum tests. Relative abundance of facultative anaerobes in healed or unhealed wounds are compared by two-sided Wilcoxon signed-rank tests (c). Taxonomic boxplots of genera with average relative abundance >0.1% (d) and taxonomic dotplot of average relative abundance of taxa within each outcome, filtered to include taxa with >0.5% average relative abundance (Pre- & Post-debridement) (e), and average relative abundance of taxa within each sample type of each outcome, filtered to include taxa >0.5% average relative abundance (f). For a – d, lower and upper bounds of the boxes correspond to the first and third quartiles, center lines indicate the median, and whiskers extend up to 1.5 x interquartile range; any points beyond the whiskers are outliers.

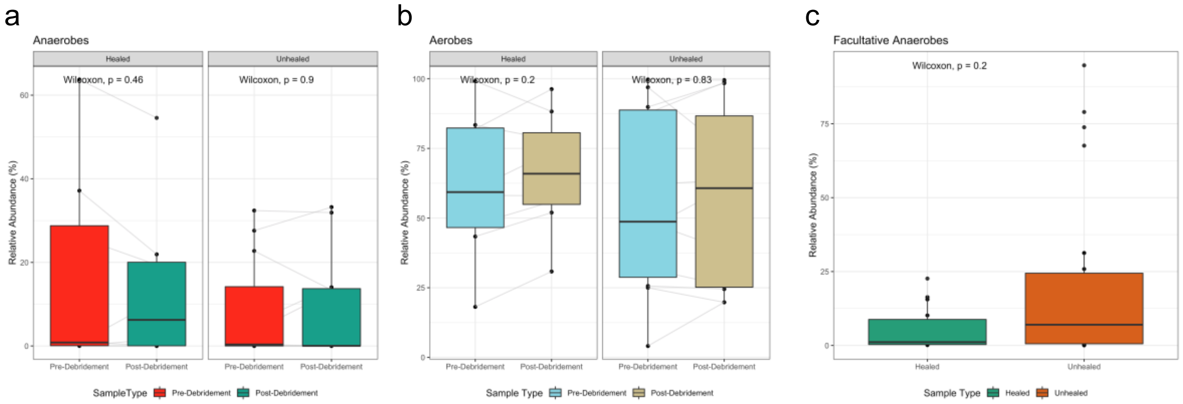

Supplementary Figure 9, continued.

d

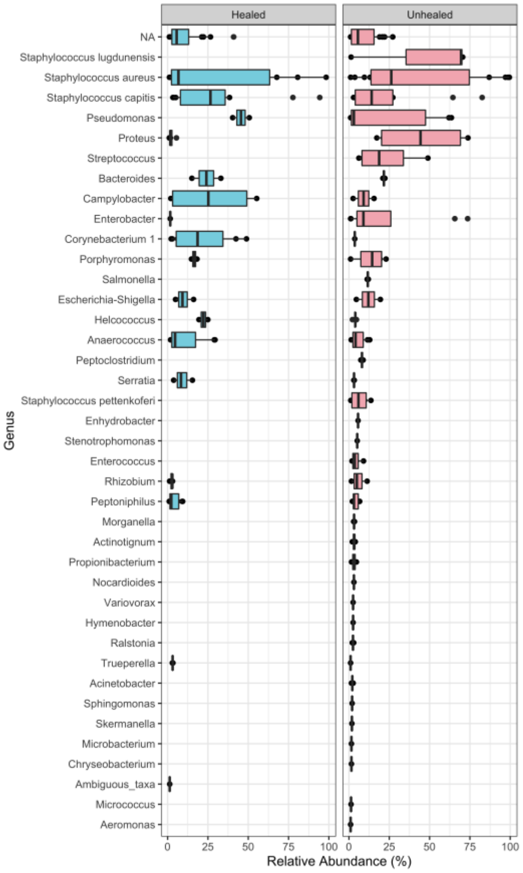

e

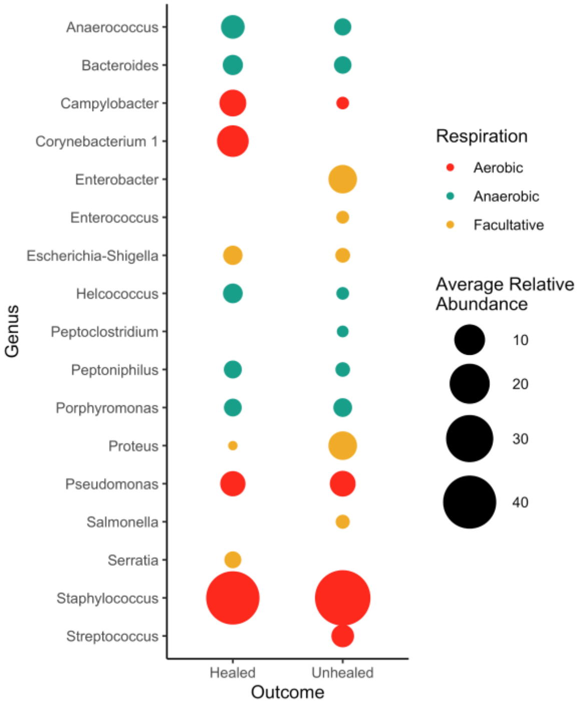

f

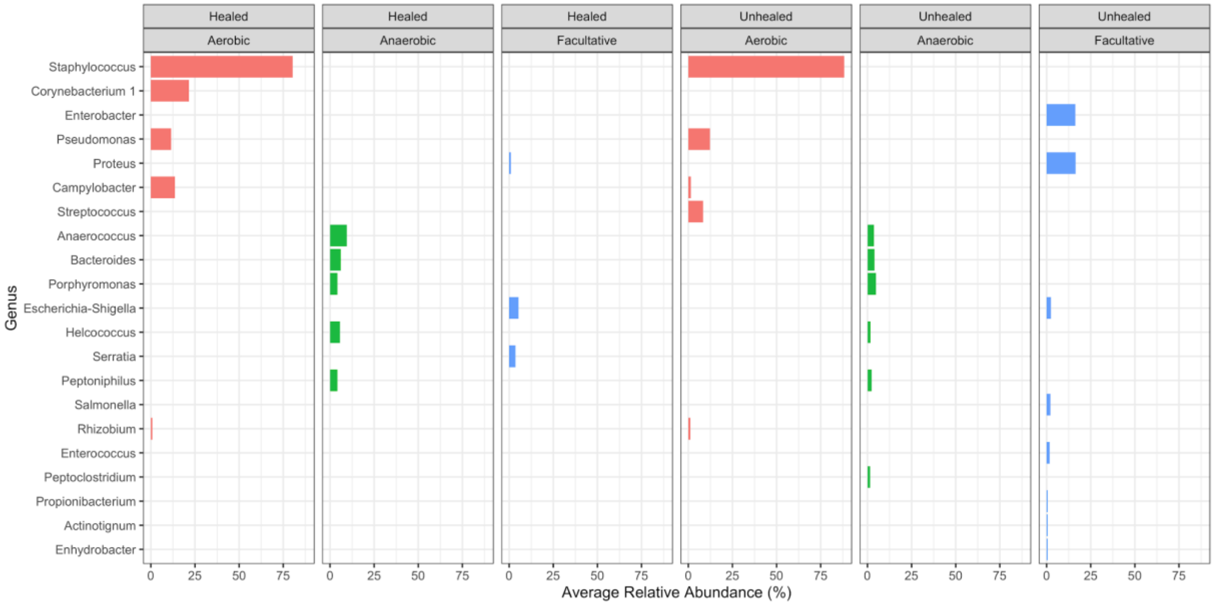

Supplementary Figure 10. Skin OTUs association with healing status, using DESeq2. OTUs with adjusted *p*-value <= 0.5 are shown and the OTU with adjusted *p*-value < 0.05 was considered as significant. Error bars indicate estimated 95% confidence intervals (1.96 x standard error, *n* = 19)

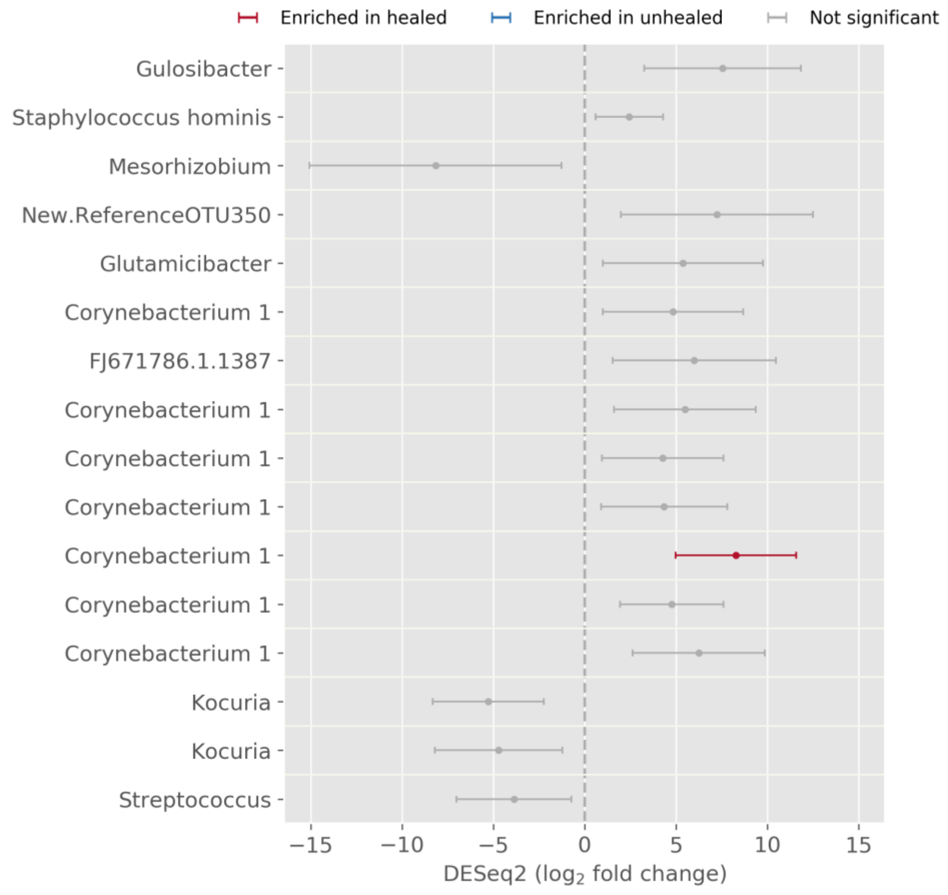

Supplementary Figure 11. Robustness of inference to exclusion of patient 16 data. Associations detected in the comparison of pre-debridement vs. skin samples in the filtered OTU table with or without patient 16 using BGLMM (a) or DESeq2 (b). Only significant OTUs with relative abundance > 0.1% are shown. Associations detected in the comparison of of pre- vs post- debridement samples in the filtered OTU table with or without patient 16 using BGLMM (c) or DESeq2 (d). Only significant OTUs in the comparison are shown. Error bars indicate indicate the 95% confidence interval (BGLMM) or estimated 95% confidence interval (DESeq2, 1.96 x standard error). With patient 16 included, n = 20; with patient 16 excluded, n = 19.

Supplementary Figure 11

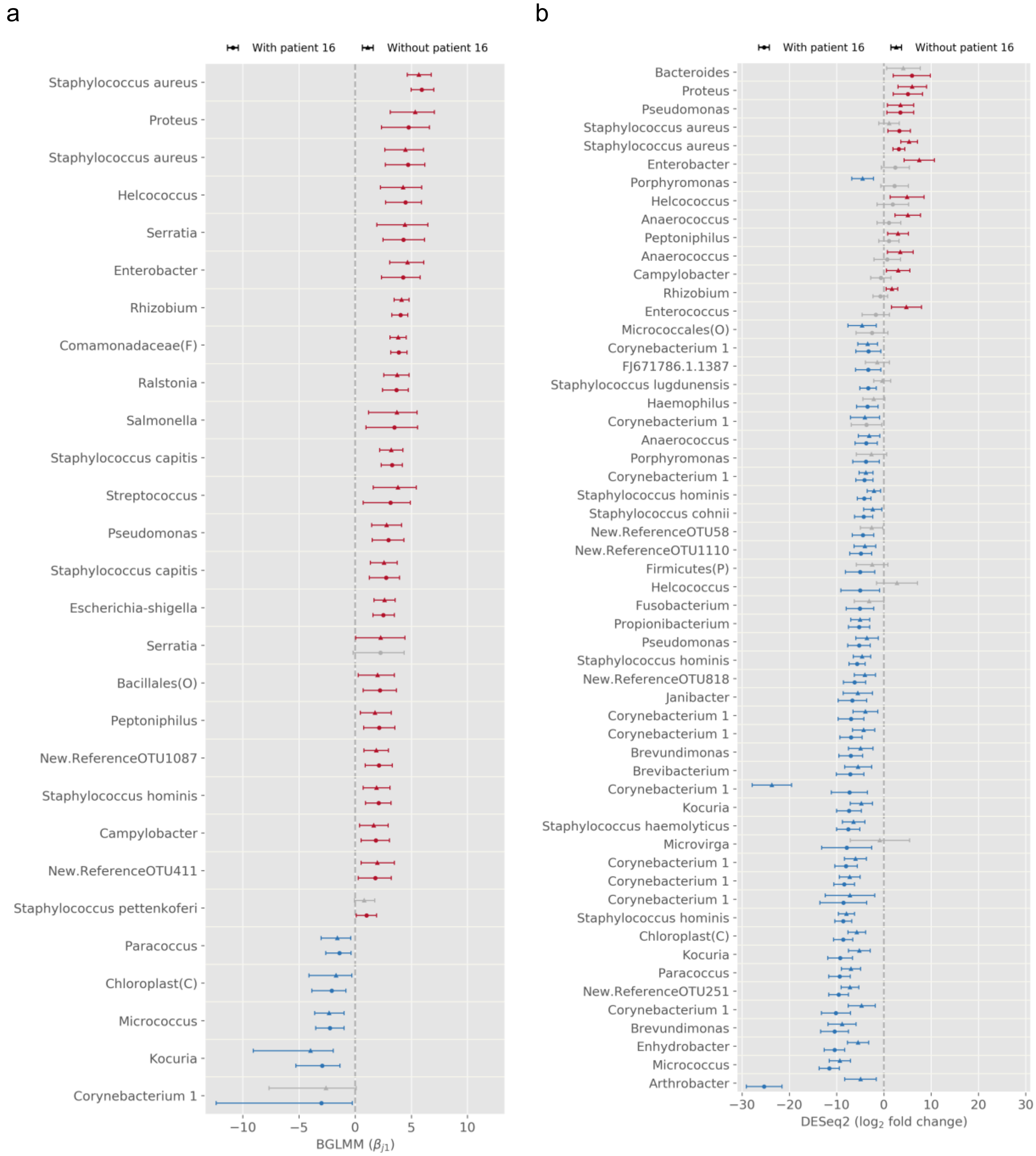

Supplementary Figure 11, continued.

c

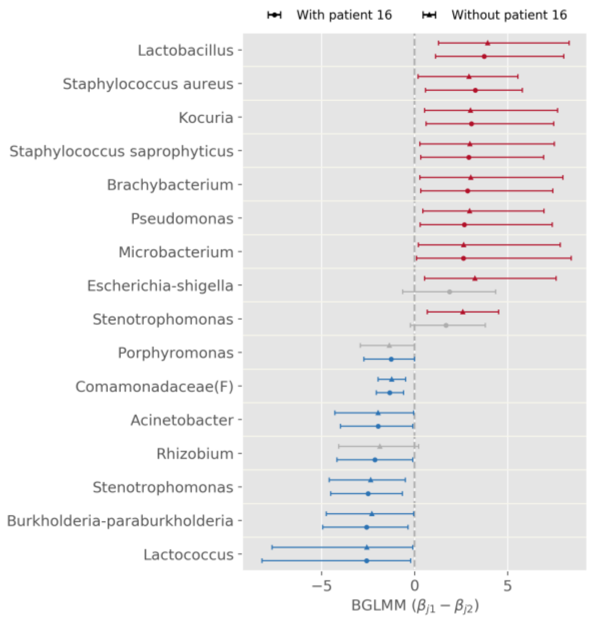

d

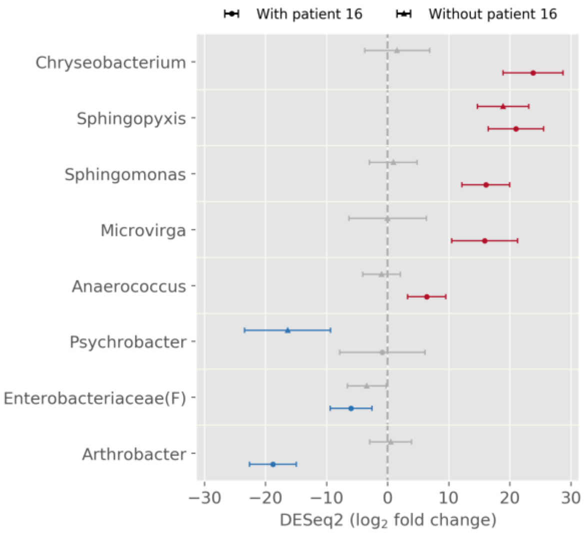

Supplementary Note 1. Further characterization of the differences between skin and wound microbiomes.

A Bray-Curtis dissimilarity matrix was calculated from the OTU table of all samples and visualized by non-metric multidimensional scaling (NMDS). This non-phylogenetic ordination showed that skin samples form a distinct cluster that is separable from wound samples (Supplementary Figure 4a). Indeed, pairwise Bray-Curtis dissimilarities between skin and wound samples were significantly different from zero (Supplementary Figure 4b). Similarly, principal coordinates analysis (PCoA) using the unweighted UniFrac distance matrix also results in clear partitioning of the skin samples from the wound samples, indicating that the skin samples share common community members across patients while the wound samples contain phylogenetically distinct taxa (Supplementary Figure 4c). Averaged across patients, pre- and post-debridement wound samples had large unweighted UniFrac distances of 0.66±0.09 (mean±standard deviation) and 0.67±0.08 to the skin sample from the same patient, respectively (Supplementary Figure 4d). However, when abundance is taken into account by PCoA ordination of the weighted UniFrac distance matrix (Supplementary Figure 4e), skin and wound samples are less distinct from each other (average weighted UniFrac distances from pre- and post-debridement wound samples to the skin sample from the same patient were 0.33±0.12 and 0.34±0.11, respectively; Supplementary Figure 4f), suggesting that high abundance taxa are shared between wound and skin while low abundance taxa distinguish skin from wound samples.

To determine the extent to which low abundance taxa were unique to skin vs. wound samples, we counted the number of OTUs in each patient that were common or unique to skin or wound samples (pre- and post-debridement combined) from that patient. On average, wound samples contained 13.7±8.7 wound-exclusive OTUs and shared 18.6±9.0 OTUs with corresponding skin samples (Supplementary Figure 4g). In contrast, skin samples contained 113±34 skin-exclusive OTUs and shared 39.9±13.1 OTUs with corresponding wound samples, indicating that most OTUs detected in a patient are exclusive to the skin while few OTUs are exclusive to the wound (Supplementary Figure 4g). However, shared OTUs were disproportionately abundant in terms of community composition, accounting for an average of 98.2±5.3% of relative abundance in wound samples and 81.7±21.5% in skin samples, emphasizing that both skin and wound samples are largely composed of shared OTUs (Supplementary Figure 4h).

Supplementary Note 2: Simulation study of BGLMM

We performed simulation studies to assess the performance of BGLMM. We assumed $J = 200$ OTUs. Like the wound microbiome dataset, the simulated data has $n = 20$ patients. It is assumed that $K = 3$ samples, a sample under each of three different experimental conditions, are collected from a patient, resulting in the covariates similar to the wound data. That is, the dataset has $P = 2$ binary covariates and $\boldsymbol{x}_{ik} = (0,0), (1,0)$ and $(0,1)$ represent the three conditions, respectively. We used the wound microbiome data to set the true values of r_{ik} and α_j. Specifically, we used the OTU counts from the dataset and computed $r'_{ik} = \log(\sum_{j=1}^{J} Y_{ijk} / \sum_{i=1}^{n} \sum_{k=1}^{K} \sum_{j=1}^{J} Y_{ijk})$ and $\alpha'_j = \log\{1/(n \times K) \sum_{i=1}^{n} \sum_{k=1}^{K} Y_{ijk}/r_{ik} + 0.01\}$. r_{ik}^{TR} were then set by randomly permuting r'_{ik} and α_j^{TR} was specified by drawing a random sample of size $J = 200$ from $\{\alpha'_j\}$. We simulated β_{jp}^{TR} from a mixture distribution that has a component concentrated at zero; $\beta_{jp}^{\text{TR}} \overset{iid}{\sim} 0.75\text{I}(\beta = 0) + 0.125\text{N}(-1.5, 0.5^2) + 0.125\text{N}(1.5, 0.5^2)$. That is, $\beta_{jp}^{\text{TR}} = 0$ (no effect) with probability 0.75. If $\beta_{jp}^{\text{TR}} \neq 0$, it was generated from either $\text{N}(-1.5, 0.5^2)$ or $\text{N}(1.5, 0.5^2)$ with equal probability. We also generated subject random effects $u_{ij} \overset{iid}{\sim} \text{N}(0, 0.5^2)$ and overdispersion parameters $s_j^{\text{TR}} \overset{iid}{\sim} \text{Gamma}(1, 10)$. We finally simulated OTU counts $Y_{ikj} \overset{indep}{\sim} \text{NB}(\mu_{ikj}^{\text{TR}}, s_j^{\text{TR}})$, where $\mu_{ikj}^{\text{TR}} = \exp(r_{ik}^{\text{TR}} + \alpha_j^{\text{TR}} + u_{ij}^{\text{TR}} + \sum_{p=1}^{P} \beta_{jp}^{\text{TR}} x_{ikp})$. Note that r_{ik}^{TR} and α_j^{TR} were specified arbitrarily using empirical values, and the distribution used to simulate β_{jp}^{TR} is different from the assumed model. We fitted the model similar to the wound data and the posterior inference is shown in Supplemental Figure 12. In panels (a) and (b) of the figure, the posterior median estimates $\hat{\beta}_{jp}$ of the regression coefficients are reasonably close to their truth β_{jk}^{TR}, and the 95% posterior credible intervals (dashed blue lines) well capture β_{jk}^{TR}. It indicates that the model well recovers the true regression coefficients and does a good job of capturing associations between the covariates and OTU abundances. We also checked the model fit using the posterior predictive distributions of Y_{ijk}. Supplemental Figure 12 (c) compares the median estimates $\hat{Y}_{ijk}^{\text{pred}}$ of the posterior predictive distributions to the observed values on the logarithmic scale. We added 0.1 to avoid numerical problems with zero. The figure indicates that the model fits the data well. We further examined the performance of our model through an additional simulation study, Simulation 2. We kept most of the simulation set-up including the specification of r_{ik}^{TR}, α_j^{TR} and u_{ij}^{TR} for Simulation 2. We assume that β_{jk}^{TR} is more sparse by generating $\beta_{jp}^{\text{TR}} \overset{iid}{\sim} 0.9\text{I}(\beta = 0) + 0.05\text{N}(-1.5, 0.5^2) + 0.05\text{N}(1.5, 0.5^2)$, i.e., on average 10% of OTUs' abundances are affected by x_{ik} in the truth. Supplemental Figure 13 illustrates that the model captures the simulation truth reasonably well, and provides a good fit to the data.

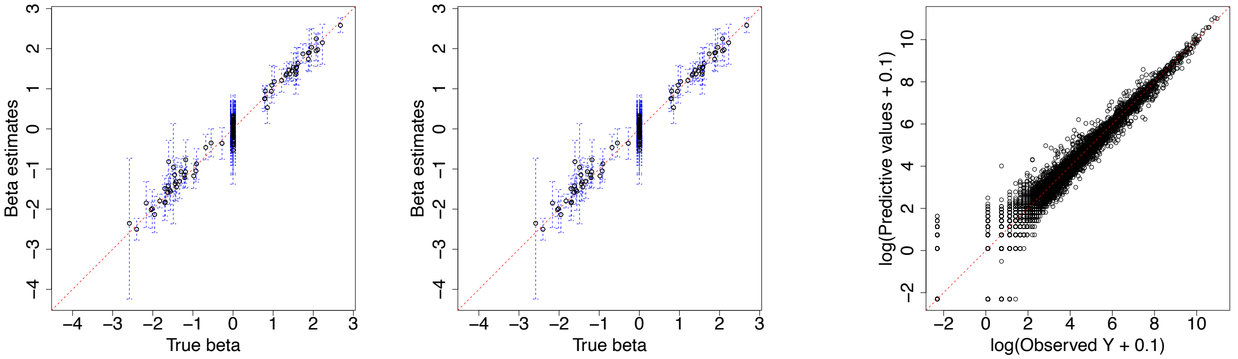

(a) $\hat{\beta}_{j1}$ vs β_{j1}^{TR} (b) $\hat{\beta}_{j2}$ vs β_{j2}^{TR} (c) $\log(Y_{ijk} + 0.1)$ vs $\log(Y_{ijk}^{\text{pred}} + 0.1)$

Figure 12: [Simulation 1] The plots in panels (a) and (b) compare posterior medians of regression coefficients $\hat{\beta}_{jk}$ to their truth, β_{jk}^{TR}. The dashed blue and solid red lines show 95% posterior credible intervals and 45 degree reference lines, respectively. In panel (c), observed counts Y_{ijk} are compared to their posterior predicted values $\hat{Y}_{ijk}^{\text{pred}}$.

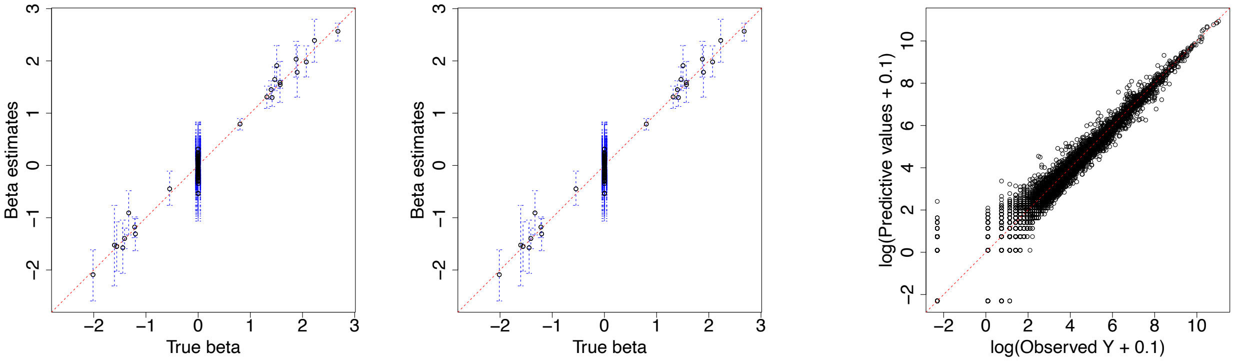

(a) $\hat{\beta}_{j1}$ vs β_{j1}^{TR} (b) $\hat{\beta}_{j2}$ vs β_{j2}^{TR} (c) $\log(Y_{ijk} + 0.1)$ vs $\log(Y_{ijk}^{\text{pred}} + 0.1)$

Figure 13: [Simulation 2] The plots in panels (a) and (b) compare posterior medians of regression coefficients $\hat{\beta}_{jk}$ to their truth, β_{jk}^{TR}. The dashed blue and solid red lines show 95% posterior credible intervals and 45 degree reference lines, respectively. In panel (c), observed counts Y_{ijk} are compared to their posterior predicted values $\hat{Y}_{ijk}^{\text{pred}}$.

Supplementary Note 3: Discussion of modeling methods

Both DESeq2 and BGLMM used a generalized linear model with a negative binomial distribution to model OTU counts, and applied shrinkage estimation to enhance prediction accuracy and interpretability of the resulting statistical inferences. However, each method employs a slightly different approach. DESeq2 takes an empirical Bayesian approach that first estimates a normal prior using maximum likelihood estimation on all OTU counts to alleviate false positive detections. It then estimates the covariate effects on abundances of individual OTUs using the empirically estimated normal prior for regression coefficients and performs statistical hypothesis tests based on the large sample approximation to infer differential abundance for fast computation. For BGLMM, we adapted the model in Lee and Sison-Magus [32] to take a fully Bayesian approach. BGLMM implements a Bayesian lasso (least absolute shrinkage and selection operator) using a Laplace prior to effectively “shrink” the regression coefficients to zero and produce improved estimates of the covariate effects. In contrast to DESeq2, which assumes the same baseline count of an OTU for all patients, BGLMM accounts for the patient-level heterogeneity in baseline counts of an OTU through patient random effects; heterogeneity of the microbiome among different chronic wounds is observed from our data, and an accommodation of the inter-patient heterogeneity may be important for our study. Indeed, BGLMM showed greater robustness to inclusion or exclusion of a single patient (Supplementary Figure 11). To fully assess the differences of each method, further computer simulation studies and extended data would be needed. Nevertheless, inferences made in agreement by both statistical techniques are likely to be robust.
